# Realistic and sustainable phosphate adsorption using intercalant-engineered exfoliated serpentinite: mechanistic insights and real-water validation

**DOI:** 10.3389/fchem.2026.1791565

**Published:** 2026-03-27

**Authors:** Haifa E. Alfassam, Amira S. Diab, Sarah I. Othman, Hanan M. Alharbi, Hassan A. Rudayni, Ahmed A. Allam, Osman Abdelghany, Mostafa R. Abukhadra

**Affiliations:** 1 Department of Biology, College of Science, Princess Nourah Bint Abdulrahman University, Riyadh, Saudi Arabia; 2 Chemistry Department, Faculty of Science, Beni-Suef University, Beni Suef, Egypt; 3 Department of Biology, College of Science, Imam Mohammad Ibn Saud Islamic University, Riyadh, Saudi Arabia; 4 Geosciences Department, College of Science, United Arab Emirates University, AlAin, United Arab Emirates

**Keywords:** exfoliated serpentinite, intercalation engineering, phosphate adsorption, real water treatment, statistical physics modeling, sustainable remediation

## Abstract

This study investigates phosphate adsorption using exfoliated serpentinite produced by intercalation with potassium nitrate (KN/SP), urea (U/SP), and potassium acetate (KC/SP), with emphasis on performance, mechanism, and realistic applicability. All modified adsorbents showed strongly improved phosphate uptake, reaching maximum capacities of 127.85 mg g^-1^ (KN/SP), 154.18 mg g^-1^ (U/SP), and 183.54 mg g^-1^ (KC/SP), confirming the advantage of acetate-assisted exfoliation. Kinetic behavior followed the pseudo-first-order model, indicating rapid, surface-controlled adsorption. Equilibrium was best fitted by the Langmuir isotherm, supporting monolayer adsorption on energetically uniform sites. Statistical physics modeling provided steric and energetic descriptors, revealing high densities of accessible sites (Nm = 39.22–63.51 mg g^-1^), with KC/SP showing the highest site density. The number of adsorbed species per site (n ≈ 4) suggests multisite/multidocking adsorption via cooperative interactions with multiple surface functionalities. Adsorption energies (<25 kJ mol^-1^) are consistent with a physisorption-dominated mechanism (electrostatic attraction, ion–dipole interactions, and hydrogen bonding), in line with the observed fast uptake and suggesting that regeneration and long-term reusability may be feasible; these latter aspects are inferred from adsorption energetics rather than directly demonstrated by extended regeneration tests in this work. Matrix effects were evaluated using competing anions (SO_4_^2-^, NO_3_^−^, HCO_3_^−^) and coexisting metals (Pb^2+^, Cu^2+^, Cd^2+^, Zn^2+^), showing only limited suppression. Validation in authentic Lake Qarun water confirmed robust phosphate removal, particularly for KC/SP, highlighting its potential as a scalable adsorbent for complex waters.

## 1 Introduction

Freshwater pollution has become a critical global challenge, threatening both ecosystem stability and human health (Yang et al., 2022; Zourou et al., 2022). The World Health Organization estimates that by 2025, more than half of the global population may face severe water scarcity, highlighting the need for effective water-quality management (Arab et al., 2022). Industrial effluents, agricultural runoff, and mining activities contribute a wide array of pollutants to aquatic systems, including pathogens, pesticides, heavy metals, fertilizers, pharmaceuticals, and synthetic dyes (Ighnih et al., 2024; Kusuma et al., 2024). Among these, phosphorus-containing species—especially phosphate ions (PO_4_^3-^)—are of particular concern because of their central role in eutrophication. Although phosphorus is vital for life, supporting essential processes such as energy transfer (Szyc et al., 2010), cellular signaling (Leclercq and Pitson, 2006), and mineral metabolism (Chande and Bergwitz, 2018), its extensive use in agriculture, detergents, and industrial applications has led to substantial phosphate accumulation in rivers and lakes (Moumen et al., 2022).

This enrichment is a principal driver of eutrophication, as even very low phosphate concentrations (as little as 0.05 mg/L) can induce rapid algal growth in stagnant or semi-enclosed waters (Gaber et al., 2025; Nguyen et al., 2024). Such blooms diminish light penetration, release toxins, and deplete dissolved oxygen during biomass decay, resulting in fish kills, biodiversity loss, and degradation of drinking-water quality (Priya et al., 2022; Yang et al., 2020). Although both nitrogen and phosphorus contribute to eutrophication, nitrogen can often be biologically removed, whereas phosphate remains considerably harder to eliminate (Schelske, 2009). Accordingly, the U.S. Environmental Protection Agency (USEPA) sets a strict limit of 25 ppb total phosphorus in surface waters to prevent these impacts (Bashar et al., 2018). Elevated phosphorus levels also pose human health risks, being linked to cardiovascular and cerebrovascular disorders, highlighting the urgent need for efficient, low-cost, and scalable phosphate-removal technologies to safeguard ecosystems and public health (Koh et al., 2019).

Several technologies are used for phosphate recovery, including adsorption (He et al., 2022; Priya et al., 2022), chemical precipitation (Deng and Dhar, 2023), ion exchange (Guida et al., 2021), biological treatment (Zhang et al., 2022), membrane filtration (Hube et al., 2020; Li et al., 2021), reverse osmosis (Zheng et al., 2023), and constructed wetlands (Kamilya et al., 2022). Chemical and biological approaches, however, often suffer from side reactions, secondary waste generation, and complex pretreatment requirements. Adsorption is therefore regarded as one of the most practical and efficient strategies for phosphate removal (Ali et al., 2019; Awad et al., 2019; Vo and Nguyen, 2024), with its performance strongly dependent on the physicochemical properties of the adsorbent that determine capacity and selectivity (Albukhari et al., 2021; Naat et al., 2021). A wide range of materials—including biochars, zeolites, LDHs, and metal oxides such as MgO, Fe_2_O_3_, CeO_2_, and Ce/Zr composites—has been investigated (Ederer et al., 2023; Khoshkalam et al., 2023; Liang et al., 2023). Selecting an effective adsorbent requires evaluating cost, synthesis ease, material availability, regeneration, kinetics, selectivity, and chemical stability (Abukhadra et al., 2020; Fan et al., 2024).

Among natural materials, clay minerals and related phyllosilicates are widely valued as adsorbents because of their abundance, low cost, and environmental friendliness (Ifa et al., 2022; Sun et al., 2024; Yang et al., 2022). They possess layered structures in which tetrahedral silicate sheets are linked to octahedral hydroxide sheets (Değermenci et al., 2022; Perelomov et al., 2025). Isomorphic substitutions—such as Al^3+^ replaced by Mg^2+^ or Fe^2+^/Fe^3+^—generate a permanent layer charge that is balanced by interlayer cations (e.g., Na^+^, Ca^2+^), resulting in the high cation-exchange capacity (CEC) central to their adsorption performance (Mejri and Oueslati, 2024; Mohan et al., 2021; Wang et al., 2024). Clays are generally classified into 1:1 types (kaolinite, serpentinite) and 2:1 types (montmorillonite, illite), all containing reactive groups (Si–OH, Al–OH, Mg–OH) that promote ion exchange and surface complexation. Their natural abundance, structural versatility, and chemical stability make them effective materials for water purification (Elmi, 2023; Mohan et al., 2021; Wang et al., 2024). To further enhance their physicochemical properties, numerous modification strategies—such as alkaline, thermal, and acid activation, metal-ion pillaring, metal-oxide incorporation, exfoliation, polymer intercalation, structural scrolling, and organic functionalization with CTAB or biopolymers—have been developed (Alomari, 2024; Barakan and Aghazadeh, 2021; Heryanto et al., 2025; Zhu et al., 2022). These treatments increase surface area, introduce new reactive groups, enlarge pore volume, and adjust surface charge, thereby improving ion exchange and creating a greater density of active adsorption sites.

Exfoliation is an effective modification strategy that delaminates layered clay minerals into nanoscale sheets, producing materials with much higher surface area, greater adsorption capacity, and improved dispersibility (Abdo et al., 2022; Ali et al., 2024). By disrupting the tightly stacked layers, exfoliation exposes previously inaccessible reactive sites, enhances ion-exchange capability, and strengthens interactions with contaminants through an increased density of active surface groups (Allah et al., 2025; Das et al., 2023). Although significant progress has been made in exfoliating and functionalizing clays, most studies have focused on common aluminosilicates such as bentonite and kaolinite because of their abundance and well-characterized structures (Ali et al., 2024; Gaber et al., 2025; Shemy et al., 2025). In contrast to widely studied aluminosilicate clays, magnesium-rich phyllosilicates such as serpentinite remain comparatively underexplored, despite their distinctive Mg-dominated crystal chemistry, which strongly influences surface reactivity, interlayer charge, and adsorption behavior (Gaber et al., 2025; Shemy et al., 2025). This unique structural framework suggests that serpentinite may undergo different modification pathways and exhibit adsorption mechanisms unlike those of conventional aluminosilicates. Therefore, further research into its exfoliation and functionalization is essential to unlock its potential for removing organic pollutants and toxic heavy metals from water (Shemy et al., 2025).

Natural serpentinite (SP, Mg_3_Si_2_O_5_(OH)_4_) is a 1:1 phyllosilicate formed through hydrothermal alteration of Mg-rich silicates such as olivine and pyroxene. It occurs in three main polymorphs—lizardite, antigorite, and chrysotile—each with distinct structural features. Structurally, serpentinite consists of an octahedral Mg–OH sheet linked to a tetrahedral Si–O sheet, with isomorphic substitutions by Fe^2+^/Fe^3+^, Ni^2+^, or Al^3+^ that influence surface charge and reactivity (Wenk et al., 2025). Abundant surface hydroxyl groups and interlayer water permit moderate adsorption through hydrogen bonding and electrostatic interactions. Nevertheless, raw serpentinite suffers from inherent limitations—including low specific surface area, restricted pore volume, and narrow interlayer spacing—that hinder the adsorption and diffusion of larger ionic or molecular species, reducing its direct applicability for chemical ions remediation (Chen et al., 2024; Shemy et al., 2025). To address these constraints, targeted chemical and physical modification strategies are essential for improving its reactivity, porosity, and adsorption performance. Among these approaches, exfoliation and delamination are especially effective; by separating the compacted layers into nanosheets, they greatly increase surface area, expose additional active sites, enhance ion-exchange capacity, and ultimately transform serpentinite into a highly efficient, low-cost, and sustainable adsorbent for wastewater treatment (Ali et al., 2024; Shemy et al., 2025).

Several exfoliation methods have been used for layered clay minerals, including high-pressure extrusion, ultrasonic sonication, chemical intercalation, and mechanical grinding (Ali et al., 2024; Zheng and Lee, 2022). Among them, chemical intercalation is considered one of the most effective approaches, as it disrupts the interlayer hydrogen-bonding network and greatly improves structural dispersion (Allah et al., 2025; Zuo et al., 2017). A wide range of intercalants—such as N-methylformamide, dimethyl sulfoxide, urea, alkylamines, potassium acetate, formamide, fatty acids, quaternary ammonium salts, and hydrazine hydrate—has been explored (Makó et al., 2019; Zuo et al., 2017). During intercalation, guest molecules penetrate the interlayer galleries, expanding basal spacing by weakening the hydrogen bonds that hold adjacent sheets together, thereby promoting delamination, improving nanosheet dispersion, and increasing accessible surface area and reactivity (Abdo et al., 2022; Makó et al., 2019). Consequently, the structural, morphological, and adsorption properties of exfoliated serpentinite, kaolinite, and other layered clays depend strongly on the chemical nature, size, and polarity of the chosen intercalants (Diab et al., 2025a; Shemy et al., 2025; Zuo et al., 2017).

To the best of our knowledge, previous studies on mineral-based phosphate adsorbents—including serpentinite and other clay minerals—have not systematically examined how the type of chemical intercalant controls intercalation-induced exfoliation and how these structural changes translate into differences in adsorption performance. In this work, we advance beyond earlier serpentinite modifications by using three distinct, simple, and scalable intercalants (CH_3_COOK, urea, KNO_3_) to engineer the interlayer chemistry, surface reactivity, and structural accessibility of magnesium-rich serpentinite, and by quantitatively linking the resulting exfoliation characteristics to adsorption-site density, ion-binding stoichiometry, and adsorption energetics through statistical-physics modeling.

Beyond this structure–mechanism analysis, the present study further extends typical mineral-based phosphate adsorption research by (i) systematically evaluating performance under competitive multi-ion conditions (SO_4_^2-^, NO_3_^−^, HCO_3_^−^, Pb^2+^, Cu^2+^, Cd^2+^, Zn^2+^), and (ii) demonstrating practical efficacy in a realistic remediation scenario using chemically complex, highly saline Lake Qarun water. Together, these elements provide a coherent structure–performance framework for intercalation-exfoliated serpentinite and establish its practical feasibility as a low-cost adsorbent for phosphate removal from complex environmental waters.

## 2 Experimental work

### 2.1 Materials

The serpentine powder (SP) used in this study was sourced from a serpentine deposit in the Eastern Desert of Egypt. Potassium acetate (CH_3_COOK, ≥98% purity), urea (99% purity), and potassium nitrate (KNO_3_, ≥98% purity) were purchased from Sigma-Aldrich and employed directly as exfoliation reagents without further purification. A certified 1,000 mg/L standard phosphate stock solution, also obtained from Sigma-Aldrich, was used for the adsorption studies. Sodium hydroxide (NaOH) pellets and nitric acid were applied for pH adjustment during synthesis. All experimental solutions were prepared using high-purity deionized water to prevent interference from extraneous ions or impurities.

### 2.2 Exfoliation of serpentine

#### 2.2.1 Exfoliation using potassium acetate (CH_3_COOK) (KC/SP)

**FIGURE 1:**
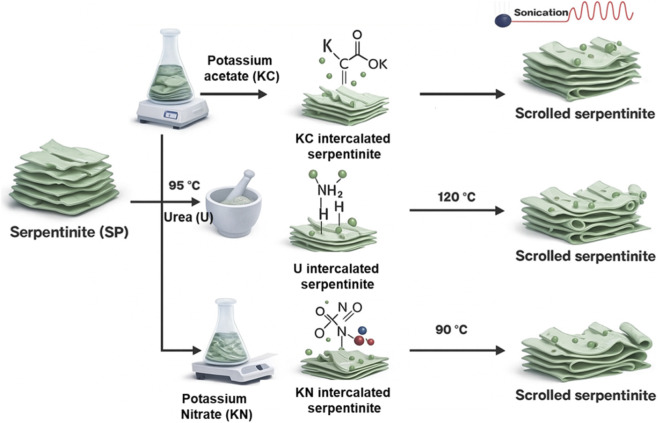
Schematic of serpentinite exfoliation and its conversion to scrolled serpentinite *via* potassium acetate, urea, and potassium nitrate treatments.

The exfoliation process was carried out following the methodology described by (Diab et al., 2025b). Raw serpentinite was first subjected to high-energy ball milling for 6 hours to obtain fine particles with sizes ranging from 20 to 100 μm. A total of 15 g of the milled material was then dispersed in 100 mL of a 4 M aqueous potassium acetate (CH_3_COOK) solution. The mixture was magnetically stirred for 12 h to ensure full hydration of the particles and effective ion exchange between acetate ions and the interlayer species within the serpentine structure. To further improve exfoliation and promote the breakdown of interlayer hydrogen bonds, the suspension was subsequently treated for an additional 48 h using a combined system of magnetic stirring and ultrasonic irradiation (240 W). The joint action of mechanical mixing and ultrasonic cavitation facilitated efficient delamination of the serpentine layers, ultimately yielding exfoliated serpentinite, as illustrated in Figure 1.

#### 2.2.2 Exfoliation by urea (U/SP)

The urea-assisted exfoliation followed the procedure reported in (Diab et al., 2025a). In brief, 6 g of finely milled serpentinite was thoroughly mixed with 3 g of urea in an agate mortar for 15 min to ensure uniform blending. The mixture was then heated at 95 °C for 48 h in a programmable muffle furnace to facilitate urea intercalation within the layered silicate structure. After heating, the solid was recovered by filtration and washed sequentially with distilled water (three cycles, 10 min each), followed by an ethanol rinse to remove unreacted urea. The cleaned material was dried at 60 °C for 24 h and subsequently subjected to an additional thermal activation at 120 °C for 1 h to promote further expansion and separation of the silicate layers. This post-treatment step yielded the exfoliated serpentinite product, designated as U/SP (Figure 1), which was stored for subsequent characterization and application studies.

#### 2.2.3 Exfoliation by KNO_3_ (KN/SP)

KNO_3_-modified serpentine was prepared by dispersing 10 g of pre-milled serpentine powder (SP) in 120 mL of an aqueous potassium nitrate solution according to the reported procedures by Diab et al. (2025b). The suspension was stirred at 500 rpm for 48 h at 90 °C to enhance ion exchange and partially disrupt the interlayer bonds of the serpentine structure. After treatment, the solid was collected by vacuum filtration and washed three times with distilled water (10 min each) to remove excess nitrate ions, followed by an ethanol rinse to improve surface cleaning. The washed material was dried at 60 °C for 24 h to retain the partially delaminated morphology. To promote further exfoliation and increase separation of the silicate sheets, the sample was subjected to an additional thermal step at 120 °C for 60 min (Figure 1). The resulting exfoliated serpentine was stored under desiccated conditions for subsequent structural characterization and adsorption experiments.

#### 2.2.4 Adsorption studies

##### 2.2.4.1 Batch adsorption studies

The phosphate uptake performance of KC/SP, U/SP, and KN/SP was examined using a series of batch adsorption experiments conducted under controlled conditions. The effects of pH (3–8), initial phosphate concentration (25–250 mg/L), and contact time (30–720 min) were systematically investigated. In all trials, the adsorbent dosage and solution volume were kept constant at 0.2 g/L and 100 mL, respectively. Temperature-dependent adsorption behavior was further assessed by performing experiments at 303–323 K using an orbital shaker incubator with precise digital temperature control to ensure uniform mixing. After each adsorption cycle, the remaining phosphate concentration was quantified using a Dionex DX-120 ion chromatography device. The PO_4_^3-^ standard that was utilized during the measurement procedures were purchased from Merck Company (Germany) and then certified by the National Standard and Technology Institute (NIST). The adsorption capacities of KC/SP, U/SP, and KN/SP were calculated using Equation 1, which incorporates the solution volume (*V* in *mL*), adsorbent mass (*m* in *mg*), and the initial (*C*_*0*_ in *mg/L*) and equilibrium (*C*_*e*_ in *mg/L*) phosphate concentrations.

$$Q_{e}=\frac{\left(C_{O}-C_{e}\right)V}{m}$$ (1)

##### 2.2.4.2 Theoretical traditional and advanced equilibrium studies

To characterize the adsorption mechanism, the experimental data were fitted to a combination of classical kinetic models, conventional isotherm equations, and advanced statistical-physics-based equilibrium models, as outlined in Supplementary Table S1. Nonlinear regression was applied to the kinetic and classical isotherm models using their respective mathematical formulations. The goodness of fit for these models was assessed using the coefficient of determination (*R* ^*2*^, Equation 2) and the Chi-square test (*χ* ^*2*^, Equation 3). For the advanced equilibrium models, predictive accuracy and model reliability were evaluated using both *R* ^*2*^ and the root mean square error (*RMSE*, Equation 4). In these expressions, *m′* denotes the total number of data points, *p* represents the number of fitted parameters, Qi,_cal_ is the model-predicted adsorption capacity, and Qi,_exp_ is the corresponding experimental value.

$$R^{2}=1-\frac{\sum {\left(Q_{e,\exp}-Q_{e,cal}\right)}^{2}}{\sum {\left(Q_{e,\exp}-Q_{e,mean}\right)}^{2}}$$ (2)

$$\chi^{2}=\sum \frac{{\left(Q_{e,\exp}-Q_{e,cal}\right)}^{2}}{Q_{e,\text{cal}}}$$ (3)

$$\text{RMSE}=\sqrt{\frac{\sum_{i=1}^{m}{\left({\text{Qi}}_{\text{cal}}-{\text{Qi}}_{\exp}\right)}^{2}}{m^{\prime }-p}}$$ (4)

## 3 Results and discussion

### 3.1 Effect of pH

**FIGURE 2:**
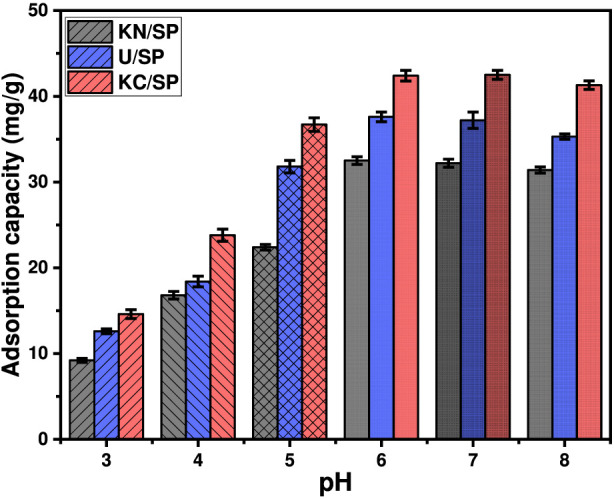
The experimental effect of the solutions pH on the adsorption of phosphate anions by KN/SP, U/SP, and KC/SP (50 mg/L concentration; 0.2 g/L dosage, 100 mL volume; and 120 min contact time).

Solution pH plays a crucial role in controlling adsorption processes because it simultaneously governs the surface charge of the adsorbent and the speciation of phosphate ions. In this study, the effect of pH on phosphate adsorption by KN/SP, U/SP, and KC/SP was examined over a pH range of 3–8 using batch experiments conducted with 50 mg/L initial phosphate concentration, 0.2 g/L adsorbent dosage, and 120 min of contact time. The adsorption trends presented in Figure 2 show a consistent increase in phosphate uptake from pH 3 to 6, a plateau at pH 7, and a slight decrease at pH 8. At pH 3, the adsorption capacities were relatively low—9.2 mg/g for KN/SP, 12.6 mg/g for U/SP, and 14.6 mg/g for KC/SP—but reached maximum values at pH 6, increasing to 31.4, 35.3, and 41.3 mg/g, respectively.

The strong dependence on pH reflects the combined influence of phosphate speciation and the surface charge characteristics of the adsorbents (Banu and Meenakshi, 2017; Gaber et al., 2025). Phosphate undergoes successive deprotonation with pKa values of 2.16, 7.20, and 10.30 for H_2_PO_4_^−^, HPO_4_^2-^, and PO_4_^3-^, respectively (Gaber et al., 2025; Zou et al., 2020). Within the pH range studied, the dominant species shift from H_2_PO_4_^−^ to HPO_4_^2-^. Since H_2_PO_4_^−^ typically forms stronger coordination and inner-sphere complexes with protonated hydroxyl and metal-binding sites than HPO_4_^2-^, adsorption is naturally favored in mildly acidic to neutral conditions (Banu et al., 2020). This behavior is reinforced by the surface charge of the adsorbents, as the measured point of zero charge (pH_PZC_) for the modified serpentine materials is approximately 8.2. Below this pH, the surfaces carry a net positive charge, enhancing electrostatic attraction toward anionic phosphate species. Above pH_PZC_, the surface becomes negatively charged and electrostatic repulsion reduces adsorption efficiency, explaining the slight decline at pH 8.

These trends highlight several important implications. First, the superior adsorption at pH 6–7 indicates that the synthesized materials operate efficiently under conditions commonly encountered in natural waters and municipal wastewater streams, reducing the need for pH adjustment and lowering treatment costs. Second, the differences in adsorption capacity among KN/SP, U/SP, and KC/SP emphasize how the exfoliation chemistry—particularly acetate-assisted delamination—governs the density and accessibility of active sites. Finally, the overall pH-dependent behavior confirms that the interplay between phosphate speciation and surface charge is the primary mechanism dictating adsorption, and it underscores the thermodynamic favorability of phosphate removal near neutral pH. These findings collectively demonstrate the practical applicability of the modified serpentinite materials in real-world phosphate remediation systems while providing mechanistic insight into their enhanced performance.

### 3.2 Kinetic studies

#### 3.2.1 Effect of contact time

**FIGURE 3:**
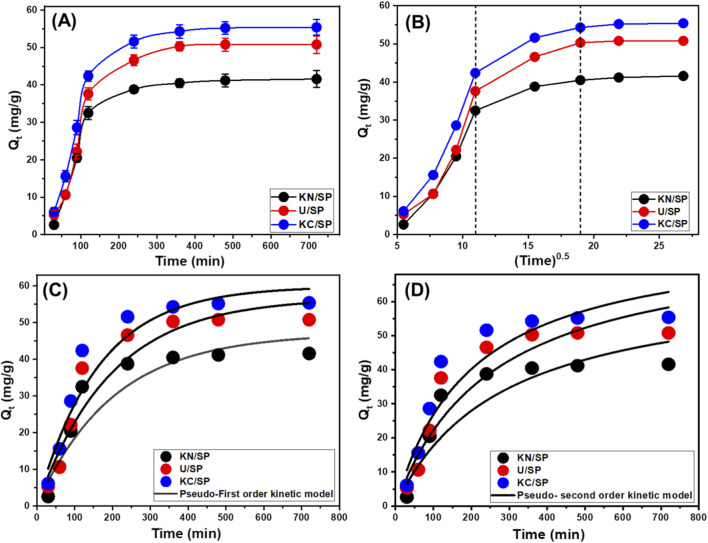
The influence of contact period on the uptake efficiencies of phosphate anions by KN/SP, U/SP, and KC/SP **(A)**, the intra-particle diffusion behaviors **(B)**, kinetic fitting with the First-order model **(C)**, and the kinetic fitting with the second-order kinetic model **(D)** (50 mg/L concentration; 0.2 g/L dosage, 100 mL volume; and pH 6).

A time-dependent adsorption study was conducted to elucidate the kinetic behavior of phosphate uptake by KN/SP, U/SP, and KC/SP under well-controlled conditions (50 mg/L initial concentration, pH 6, 30 °C, 100 mL solution volume, and 0.2 g/L adsorbent dosage). By varying the contact time from 30 to 720 min, the temporal evolution of adsorption was monitored to better understand the interaction dynamics between phosphate ions and the functionalized serpentinite surfaces (Figure 3A). The adsorption profiles revealed a rapid phase of phosphate removal during the initial period, followed by a gradual decline in adsorption rate until equilibrium was reached at approximately 360 min. At equilibrium, the uptake capacities were 40.5 mg/g for KN/SP, 50.3 mg/g for U/SP, and 54.3 mg/g for KC/SP, confirming that all materials were effective, though with clear performance differences (Figure 3A).

The pronounced initial uptake is characteristic of systems with abundant, energetically favorable active sites, where phosphate ions can readily interact with surface hydroxyl groups, exposed magnesium centers, and newly created adsorption sites generated during exfoliation (El-Sherbeeny et al., 2021). This stage corresponds to a period dominated by film diffusion and rapid surface complexation, where the driving force for adsorption is high due to the steep concentration gradient between the bulk solution and the particle surface (Beck et al., 2021). The sharp increase in removal efficiency during this initial period indicates strong affinity between phosphate ions and the modified serpentinite structures, demonstrating the effectiveness of exfoliation in improving site accessibility.

As adsorption progresses, the availability of vacant active sites decreases, and the system transitions into a slower kinetic regime where intraparticle diffusion, steric hindrance, and repulsion between already-adsorbed species play increasingly important roles (Gaber et al., 2025). The plateau observed after 360 min signifies that the system has reached dynamic equilibrium, where the rates of adsorption and desorption become comparable. This behavior confirms that the majority of high-energy binding sites were occupied and additional uptake occurred only marginally as the system approached saturation. Such kinetic trends closely match those previously reported for phosphate adsorption onto engineered clay-based and oxide-modified materials, reinforcing the mechanism proposed here (Alhalili and Abdelrahman, 2024).

The differences among the three materials carry important mechanistic implications. KC/SP exhibited the highest equilibrium capacity, which suggests that acetate-assisted exfoliation results in more accessible surface area, higher pore connectivity, or a greater density of coordination-active functional groups compared to urea- or nitrate-treated samples. This indicates that the nature of the intercalating agent not only affects structural delamination, but also governs the formation and distribution of chemically active adsorption sites. U/SP showed intermediate performance, consistent with the ability of urea to disrupt hydrogen bonding and expand interlayer spacing, while N/SP displayed the lowest capacity, suggesting more limited exfoliation or fewer high-affinity binding sites (Cheng et al., 2010; Zhang et al., 2017; Zhang et al., 2020).

The behavior observed across the materials underscores the significance of exfoliation chemistry in tailoring adsorption performance. The rapid initial adsorption and relatively short equilibrium time reflect strong binding affinity, which is valuable for practical wastewater treatment operations that require efficient adsorption within limited contact periods. Moreover, the high equilibrium capacities demonstrate that the modified serpentinite structures are capable of sustaining significant adsorptive loading, a critical requirement for real-world applications where phosphate concentrations can fluctuate widely.

#### 3.2.2 Intra-particle diffusion behavior

The adsorption mechanism of phosphate ions onto KN/SP, U/SP, and KC/SP was further examined using intra-particle diffusion analysis, providing deeper insight into the sequence of mass-transfer steps governing phosphate uptake. As shown in Figure 3B, the diffusion plots exhibit three distinct linear regions, each characterized by different slopes, indicating that adsorption does not occur through a single homogeneous process but instead proceeds through multiple sequential steps along the diffusion pathway (El Qada, 2020; Gaber et al., 2025). The first linear segment, corresponding to the initial stage, features a steep slope and represents rapid external adsorption or boundary-layer diffusion (Figure 3B). During this phase, phosphate ions interact predominantly with readily accessible active sites on the outer surfaces of the exfoliated serpentinite materials. This rapid uptake reflects the strong affinity of phosphate species for the positively charged or functionally enriched surface groups present on the modified adsorbents (Albukhari et al., 2021; Lin et al., 2021).

The second linear region is characterized by a reduced slope, signifying the transition to intra-particle diffusion as the dominant rate-controlling mechanism (Figure 3B). Once the external surface sites become progressively occupied, phosphate ions begin migrating into the internal pores and interlayer spaces of the exfoliated structures. This stage reflects the influence of pore geometry, layer separation, and the degree of exfoliation on the adsorbent’s ability to transport ions into its interior, where diffusion resistance becomes more pronounced (El Qada, 2020; Lin et al., 2021). The third region of the plot trends toward a plateau, representing the final phase of adsorption where the system approaches equilibrium (Figure 3B). In this stage, the rate of phosphate uptake decreases substantially as high-energy binding sites are depleted and only limited, lower-affinity sites remain available. Adsorption becomes increasingly governed by slower molecular-level interactions, including weak electrostatic forces, surface complexation, and steric limitations within the confined pore structure (Abukhadra et al., 2022).

The presence of these multi-linear regions confirms that phosphate adsorption onto the exfoliated serpentine materials follows a complex, multi-stage kinetic mechanism involving both external surface adsorption and subsequent intra-particle diffusion. This mechanistic interpretation underscores the importance of exfoliation, which enhances pore accessibility and surface reactivity, thereby facilitating efficient transport of phosphate ions from the boundary layer into the internal structure of the adsorbent before final site saturation occurs. These findings align with the fundamental diffusion theory for porous materials and further validate the structural advantages introduced by the applied modification routes.

#### 3.2.3 Kinetic modeling

Kinetic modeling is a fundamental tool for interpreting adsorption behavior because it provides insight into the rate-controlling steps that govern how quickly and efficiently pollutants interact with an adsorbent surface. Two of the most widely applied kinetic models in adsorption science are the pseudo-first-order (PFO) and pseudo-second-order (PSO) models, each rooted in distinct mechanistic assumptions. The PFO model, originally derived from Lagergren’s rate law, describes systems where the adsorption rate is proportional to the number of unoccupied sites and is often consistent with surface-controlled or physisorption-dominated uptake; however, similar PFO behavior can also arise in mixed or diffusion-influenced processes (Rocky et al., 2026; Vareda, 2023). In contrast, the PSO model is based on the assumption that chemisorption is the dominant mechanism, involving stronger valence interactions—electron sharing, covalent bonding, or ligand exchange—that typically require higher activation energies and proceed at slower rates (Tran, 2023; Wang and Guo, 2020).

In the present study, the adsorption kinetics of phosphate ions onto KN/SP, U/SP, and KC/SP were analyzed using both models to better elucidate the mechanistic nature of phosphate uptake. Nonlinear regression was employed to fit the experimental data, and model accuracy was assessed using the coefficient of determination (*R* ^*2*^) and the Chi-squared error (*χ* ^*2*^), as presented in Table 1 and Figures 3C,D. Across all samples, the PFO model demonstrated superior performance, consistently showing higher R^2^ values and significantly lower *χ* ^*2*^ values compared to the PSO model. This strong statistical adherence indicates that phosphate adsorption onto the modified serpentinite surfaces is more accurately described by PFO kinetics. This conclusion is reinforced by the strong agreement between the experimentally measured equilibrium adsorption capacities and those predicted by the PFO model. Experimentally, the equilibrium capacities were 41.6 mg/g for KN/SP, 50.8 mg/g for U/SP, and 55.4 mg/g for KC/SP, while the PFO model predicted values of 46.99 mg/g, 56.74 mg/g, and 59.89 mg/g, respectively (Table 1). The close correspondence between these values strongly supports the prevalence of physisorption as the dominant mechanism controlling the adsorption process (Hassan et al., 2022).

**TABLE 1:** The estimated theortical parameters of the assessed kinetic models.

Adsorbent	Model	Parameter	Values
KN/SP	**Pseudo-first order**	***K***_***1***_ ***(1/min)*** ***Q***_***e***_ ***(mg/g)*** ***R*** ^***2***^ ***X*** ^***2***^	0.00494 46.99 0.89 2.01
	**Pseudo-second order**	***K***_***2***_ ***(mg/g min)*** ***Qe (mg/g)*** ***R*** ^***2***^ ***X*** ^***2***^	5.33 × 10^−5^ 67.017 0.87 2.39
U/SP	**Pseudo-first order**	***K***_***1***_ ***(1/min)*** ***Q***_***e***_ ***(mg/g)*** ***R*** ^***2***^ ***X*** ^***2***^	0.00515 56.74 0.92 1.39
	**Pseudo-second order**	***K***_***2***_ ***(mg/g min)*** ***Q***_***e***_ ***(mg/g)*** ***R*** ^***2***^ ***X*** ^***2***^	4.96 × 10^−5^ 78.97 0.90 1.77
KC/SP	**Pseudo-first order**	***K***_***1***_ ***(1/min)*** ***Q***_***e***_ ***(mg/g)*** ***R*** ^***2***^ ***X*** ^***2***^	0.00621 59.89 0.93 1.21
	**Pseudo-second order**	***K***_***2***_ ***(mg/g min)*** ***Q***_***e***_ ***(mg/g)*** ***R*** ^***2***^ ***X*** ^***2***^	6.24 × 10^−5^ 80.27 0.91 1.67

The weaker performance of the PSO model does not exclude the possibility of chemical interactions; rather, it suggests that chemisorption plays a secondary or complementary role (Diab et al., 2025a). This interpretation is consistent with previous reports showing that phosphate adsorption onto clay-based or layered mineral adsorbents may involve localized chemisorptive interactions—such as hydrogen bonding, surface complexation, or specific ligand exchange—followed by extensive physical adsorption as the surface becomes increasingly populated (Abukhadra et al., 2024; Shemy et al., 2025). Such behavior points toward a hybrid or multi-mechanistic adsorption pathway, where an initial chemisorptive layer may serve as an anchoring platform for subsequent physisorption, resulting in an overall kinetic profile more closely aligned with the PFO model (Bakalis and Zerbetto, 2025; Vareda, 2023).

Taken together, these findings demonstrate that phosphate removal by the exfoliated serpentinite materials proceeds primarily through fast, diffusion-driven physical adsorption, supported to a lesser extent by localized chemical interactions. This is an important result for practical applications: physisorption-dominated processes are typically faster, more reversible, and require lower regeneration energy, making the adsorbents more suitable for large-scale wastewater treatment operations where efficiency and reusability are critical. The enhanced performance observed for KC/SP, followed by U/SP and KN/SP, further highlights the importance of exfoliation chemistry in optimizing adsorption kinetics by increasing the accessibility and density of active sites.

### 3.3 Equilibrium studies

#### 3.3.1 Effect of initial concentrations

**FIGURE 4:**
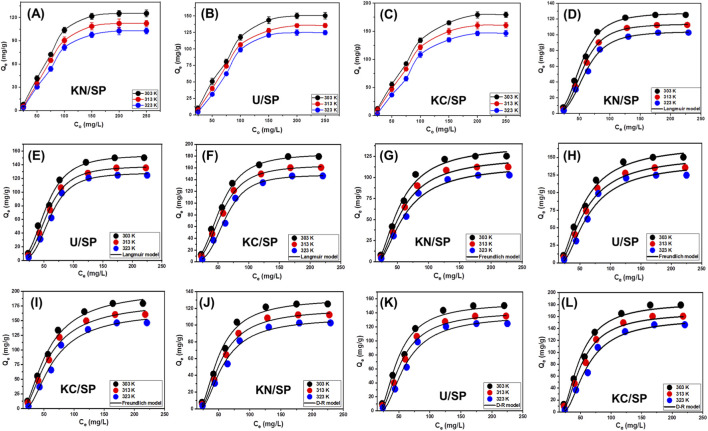
Shows the experimental effect of the starting phosphate concentrations on the uptake behaviors (KN/SP **(A)**, U/SP **(B)**, and KC/SP **(C)**) and fitting of the phosphate adsorption properties with classic isotherm models including Langmuir model (KN/SP **(D)**, U/SP **(E)**, and KC/SP **(F)**), Freundlich model (KN/SP **(G)**, U/SP **(H)**, and KC/SP **(I)**), and D-R model (KN/SP **(J)**, U/SP **(K)**, and KC/SP **(L)**) (24 h contact time; 0.2 g/L dosage, 100 mL volume; and pH 6).

The influence of initial phosphate concentration on adsorption performance was examined for KN/SP, U/SP, and KC/SP in order to determine their maximum adsorption capacities and equilibrium characteristics over a concentration range of 25–250 mg/L. All experiments were conducted under controlled conditions, using 100 mL of solution, an adsorbent dosage of 0.2 g/L, a 24-h contact time, and temperatures of 303 K, 313 K, and 323 K. As shown in Figures 4A–C, increasing the initial phosphate concentration resulted in a progressive rise in adsorption capacity for all materials. This behavior reflects the increased mass-transfer driving force at higher solute concentrations, which enhances the probability of collision and interaction between phosphate ions and the available adsorption sites on the modified serpentinite surfaces (Alphonce et al., 2025; Zhao et al., 2025). However, at higher concentrations the adsorption curves gradually approached a plateau, indicating the saturation of active sites and the establishment of adsorption equilibrium. The maximum equilibrium capacities for KN/SP were 125.3 mg/g at 303 K, 112.4 mg/g at 313 K, and 102.7 mg/g at 323 K (Figure 4A). U/SP exhibited substantially higher capacities of 150.4 mg/g, 135.5 mg/g, and 124.7 mg/g at the corresponding temperatures (Figure 4B). Among all materials, KC/SP achieved the highest adsorption capacities—179.4 mg/g at 303 K, 160.6 mg/g at 313 K, and 146.3 mg/g at 323 K (Figure 4C).

The superior performance of KC/SP can be attributed to the structural advantages conferred by acetate-assisted exfoliation, which enhances specific surface area, increases the number of exposed reactive sites, and improves pore accessibility (Cheng et al., 2010; Diab et al., 2025b). These features collectively promote stronger interactions between phosphate species and the modified serpentinite surface, enabling higher binding capacities. In contrast, U/SP and KN/SP—although effective—exhibit comparatively fewer high-energy binding sites, consistent with their lower degrees of exfoliation and structural activation. A notable trend observed in all three materials is the decline in adsorption capacity with increasing temperature. This decrease indicates that phosphate adsorption onto the exfoliated serpentinite systems is exothermic, meaning that lower temperatures favor the formation of adsorbate–adsorbent interactions. The negative temperature dependence is consistent with previously reported thermodynamic behavior for phosphate adsorption on nanostructured clay-based and oxide-modified materials, where rising temperature reduces the stability of surface complexes and weakens electrostatic attractions (Kalaitzidou et al., 2022; Nadagouda et al., 2024).

Overall, the equilibrium results highlight two key conclusions: (i) the adsorption process is strongly concentration-dependent and driven by both surface-site availability and mass-transfer forces, and (ii) acetate-modified serpentinite exhibits a clear advantage in adsorptive performance due to its enhanced structural features. These findings provide valuable insights into the practical applicability of the materials, demonstrating that KC/SP, in particular, offer high capacity at relatively low temperatures—conditions favorable for real wastewater treatment systems.

#### 3.3.2 Giles’s classification

The equilibrium adsorption profiles of phosphate on KN/SP, U/SP, and KC/SP were further analyzed using Giles’s classification framework, which provides a qualitative interpretation of isotherm shapes and their underlying adsorption mechanisms. According to the patterns displayed in Figures 4A–C, all three materials exhibit characteristic L-type isotherms, a class commonly associated with strong adsorbate–adsorbent affinity and progressive monolayer formation (Giles et al., 1960). L-type behavior typically arises when the first molecules of the adsorbate display a high degree of attraction toward the adsorbent surface, leading to rapid site occupation at low concentrations and gradually decreasing adsorption rates as surface sites become filled (Abukhadra et al., 2018; Giles et al., 1960).

The appearance of L-type isotherms in this study indicates that phosphate ions interact strongly with the functional groups exposed on the exfoliated serpentinite surfaces. This is consistent with the presence of abundant reactive sites—such as surface hydroxyl groups, Mg-centered coordination sites, and defect-generated adsorption domains—that were created or enhanced during the exfoliation and modification processes. The shape of the isotherms also implies that the active binding sites on KN/SP, U/SP, and KC/SP are distributed relatively uniformly, enabling consistent adsorption behavior across the surface until saturation is reached (Abukhadra et al., 2018). At lower phosphate concentrations, the strong upward curvature of the L-type profiles reflects highly favorable adsorption due to the dominance of high-energy surface sites. As the concentration increases, the gradual leveling of the curves indicates the formation of a monolayer, consistent with a finite number of available adsorption sites—a key assumption of monolayer-dominated systems. This behavior aligns with the structural characteristics of the modified serpentinite materials, which offer a high density of accessible coordination and electrostatic binding sites without significant multilayer formation or cooperative interactions.

The clear L-type signature observed for all materials underscores their high affinity toward phosphate, with KC/SP showing the most pronounced behavior. This further confirms the enhanced adsorptive performance of acetate-modified serpentinite, attributable to its improved surface area, pore accessibility, and surface reactivity. Collectively, the Giles’s classification results highlight the strong phosphate-binding capability of KN/SP, U/SP, and KC/SP and demonstrate their suitability as effective adsorbents for treating phosphate-rich or eutrophic wastewater streams.

#### 3.3.3 Classic isotherm models

Classical adsorption isotherm models play an essential role in understanding the equilibrium distribution of pollutants between the aqueous phase and solid adsorbents. By applying these models, it becomes possible to elucidate key aspects of the adsorption process, including the strength and nature of the adsorbate–adsorbent interaction, the capacity of the adsorbent to retain target molecules, and the theoretical limits governing monolayer or multilayer uptake (Al-Ghouti and Da’ana, 2020). In the present work, the equilibrium behavior of phosphate on KN/SP, U/SP, and KC/SP was analyzed using three widely applied isotherm models: Langmuir (Figures 4D–F), Freundlich (Figures 4G–I), and Dubinin–Radushkevich (D–R) (Figures 4J–L). Each model was fitted using its non-linear mathematical expression, as summarized in Table 2, and the fitting performance was evaluated using the coefficient of determination (R^2^) and Chi-squared (χ^2^) error values.

**TABLE 2:** The estimated theortical parameters of the assessed classic and advanaced isotherm models (24 h contact time; 0.2 g/L dosage, 100 mL volume; and pH 6).

Parameters of the classic isotherm models					
Materials	Model	Parameter	Temperature (^o^ K)		
			303 K	313 K	323 K
KN/SP	**Langmuir**	*q*_*max*_ *(mg/g)* *K*_*L*_ *(L/mg)* *R* ^*2*^ *X* ^*2*^ *R*_*L*_	127.895 2.32 × 10^−6^ 0.998 0.138 0.999	113.99 9.56 × 10^−7^ 0.999 0.051 0.999	104.25 8.28 × 10^−7^ 0.995 0.266 0.995
	**Freundlich**	*K*_*f*_ *(mg/g)* *1/n* *R* ^*2*^ *X* ^*2*^	138.81 0.51 0.993 0.389	124.68 0.502 0.995 0.273	115.12 0.512 0.994 0.312
	**D-R**	*q*_*max*_ *(mg/g)* *K*_*DR*_ *(mol* ^*2*^ */kJ* ^*2*^ *)* *E (KJ/mol)* *R* ^*2*^ *X* ^*2*^	131.66 0.764 0.809 0.990 0.537	119.25 0.877 0.755 0.993 0.370	108.76 0.988 0.711 0.992 0.434
U/SP	**Langmuir**	*q*_*max*_ *(mg/g)* *K*_*L*_ *(L/mg)* *R* ^*2*^ *X* ^*2*^ *R*_*L*_	154.38 7.48 × 10^−6^ 0.994 0.377 0.998–0.999	138.16 2.44 × 10^−6^ 0.998 0.065 0.999	128.09 4.25 × 10^−7^ 0.997 0.185 0.997
	**Freundlich**	*K*_*f*_ *(mg/g)* *1/n* *R* ^*2*^ *X* ^*2*^	168.56 0.546 0.993 0.422	153.01 0.537 0.993 0.415	143.19 0.512 0.987 0.845
	**D-R**	*q*_*max*_ *(mg/g)* *K*_*DR*_ *(mol* ^*2*^ */kJ* ^*2*^ *)* *E (KJ/mol)* *R* ^*2*^ *X* ^*2*^	154.39 0.706 0.841 0.986 0.878	142.23 0.849 0.767 0.988 0.715	136.43 1.1 0.674 0.987 0.838
KC/SP	**Langmuir**	*q*_*max*_ *(mg/g)* *K*_*L*_ *(L/mg)* *R* ^*2*^ *X* ^*2*^ *R*_*L*_	184.06 9.53 × 10^−6^ 0.997 0.210 0.997–0.999	164.41 4.41 × 10^−6^ 0.997 0.165 0.998–0.999	148.16 3.36 × 10^−7^ 0.992 0.664 0.999
	**Freundlich**	*K*_*f*_ *(mg/g)* *1/n* *R* ^*2*^ *X* ^*2*^	204.28 0.568 0.995 0.366	182.83 0.552 0.994 0.423	166.11 0.508 0.991 0.672
	**D-R**	*q*_*max*_ *(mg/g)* *K*_*DR*_ *(mol* ^*2*^ */kJ* ^*2*^ *)* *E (KJ/mol)* *R* ^*2*^ *X* ^*2*^	183.31 0.717 0.835 0.984 1.162	166.78 0.809 0.786 0.986 0.954	157.04 1.111 0.671 0.990 0.760
Steric and energetic parameters of the advanced isotherm model					
KN/SP	**Monolayer of one energy**	*n* *N*_*m*_ *(mg/g)* *Q*_*sat*_ *(mg/g)* *C*_*1/2*_ *(mg/L)* *ΔE (KJ/mol)* *R* ^*2*^ *X* ^*2*^	3.26 39.22 127.85 53.44 −21.19 0.997 0.138	3.44 33.11 113.89 56.02 −22.12 0.999 0.051	3.42 30.47 104.21 59.93 −22.28 0.996 0.256
U/SP	**Monolayer of one energy**	*n* *Nm (mg/g)* *Q*_*sat*_ *(mg/g)* *C1/2 (mg/L)* *ΔE (KJ/mol)* *R* ^*2*^ *X* ^*2*^	2.96 52.09 154.18 53.64 −21.18 0.994 0.377	3.20 43.06 137.79 56.09 −22.12 0.998 0.065	3.56 35.88 127.73 60.91 −22.95 0.997 0.185
KC/SP	**Monolayer of one energy**	*n* *N*_*m*_ *(mg/g)* *Q*_*sat*_ *(mg/g)* *C*_*1/2*_ *(mg/L)* *ΔE (KJ/mol)* *R* ^*2*^ *X* ^*2*^	2.89 63.51 183.54 54.01 −21.17 0.997 0.211	3.06 53.63 164.1 55.86 −22.22 0.997 0.165	3.61 41.05 148.19 62.16 −22.89 0.992 0.664

Among the models tested, the Langmuir isotherm consistently provided the best correlation for all three adsorbents. The strong agreement with Langmuir theory implies that phosphate adsorption occurs predominantly through monolayer coverage on a surface composed of energetically uniform binding sites (Lincold et al., 2025; Usman et al., 2022). This finding indicates that phosphate ions interact with well-defined, equivalent active sites generated by the exfoliation and surface activation of the serpentinite materials. The uniformity suggested by the Langmuir fit aligns with the structural characteristics of the modified serpentinite, where exfoliation increases surface accessibility and exposes a relatively homogeneous distribution of reactive functional groups.

In comparison, the Freundlich model—which assumes adsorption on a heterogeneous surface with varying site energies and potential multilayer formation—exhibited noticeably weaker correlation with the experimental data (Huang et al., 2018; Sherlala et al., 2019). The poorer fit suggests that the surfaces of KN/SP, U/SP, and KC/SP behave more uniformly than heterogeneous, further reinforcing the suitability of the Langmuir model. Additional confirmation of favorable adsorption behavior is provided by the Langmuir separation factor (RL), whose values were less than 1 for all materials and temperatures studied, indicating that phosphate adsorption proceeds in a favorable thermodynamic regime (Shemy et al., 2025).

The maximum monolayer adsorption capacities (Q_max_) predicted by the Langmuir model were 125.3, 112.4, and 102.7 mg/g for KN/SP at 303, 313, and 323 K, respectively. For U/SP, Qmax values reached 150.4, 135.5, and 124.7 mg/g at the same respective temperatures. KC/SP exhibited the highest monolayer capacities—179.4 mg/g at 303 K, 160.6 mg/g at 313 K, and 146.3 mg/g at 323 K—confirming its superior adsorption performance. These values are consistent with earlier structural and kinetic observations, showing that acetate-modified serpentinite possesses enhanced surface area, greater pore accessibility, and a larger population of active binding sites. Overall, the isotherm analysis highlights that monolayer adsorption dominates phosphate uptake on the exfoliated serpentinite materials, and it underscores KC/SP as the most efficient adsorbent among the three. The superior performance reflects the effectiveness of the chosen modification route and provides strong validation of these materials for high-capacity phosphate remediation applications.

The equilibrium parameters derived from the Dubinin–Radushkevich (D–R) isotherm model provide an additional layer of mechanistic understanding by describing the energetic characteristics of phosphate adsorption onto KN/SP, U/SP, and KC/SP independently of surface uniformity (Hu and Zhang, 2019; Shemy et al., 2025). Unlike models such as Langmuir or Freundlich—which infer interaction types based primarily on surface assumptions—the D–R model enables direct estimation of the mean adsorption energy (E), which serves as a sensitive indicator for differentiating between physical and chemical adsorption mechanisms (Mahanty et al., 2023; Puccia et al., 2021). The classification of adsorption based on E is well established: values below 8 kJ/mol typically correspond to physisorption driven by weak van der Waals interactions, electrostatic forces, and diffusion-mediated processes; values between 8 and 16 kJ/mol reflect mixed-mode adsorption, where both physical and chemical interactions contribute; and values greater than 16 kJ/mol signify chemisorption involving stronger valence forces, such as electron sharing, surface complexation, or ligand exchange (Diab et al., 2025b; Gaber et al., 2025).

In this study, all calculated E values for phosphate adsorption onto the three SP-based exfolaited adsorbents were consistently below 8 kJ/mol (Table 2). This uniform trend strongly indicates that the adsorption process is governed predominantly by physisorption. The low-energy nature of the interaction suggests that phosphate ions bind mainly through electrostatic attraction to positively charged or protonated sites, hydrogen bonding with surface hydroxyl groups, and weak non-covalent interactions with exfoliated layer surfaces. These findings are consistent with earlier kinetic results, where the pseudo-first-order (PFO) model showed the strongest fit, signaling a rate-controlling mechanism typical of physisorption rather than chemisorption. The convergence of D–R, PFO, and Langmuir analyses therefore reinforces a coherent mechanistic narrative: phosphate uptake occurs primarily *via* monolayer physisorption onto energetically uniform binding sites generated during the exfoliation process.

Importantly, the dominance of physisorption has several practical and scientific implications. First, physisorption-controlled systems tend to exhibit faster adsorption rates, as the process does not require the activation energies associated with chemical bond formation (Diab et al., 2025a). This observation supports the experimentally observed rapid uptake during early contact times, especially for the highly exfoliated KC/SP. Second, physisorption is generally reversible, which greatly enhances the potential for adsorbent regeneration and reusability—an essential factor in designing sustainable phosphate recovery systems (Kumari et al., 2025; Suresh Kumar et al., 2018). Third, the low-energy interactions confirm that the structural modifications introduced during exfoliation—particularly the increase in surface area, exposure of Mg–OH sites, and expansion of interlayer spacing—play a more dominant role in enhancing adsorption performance than the formation of strong chemical coordination bonds (Budnyak et al., 2020; Mobarak et al., 2021). Finally, the D–R findings highlight an important comparative advantage of the KC/SP material. Although all three SP-based exfoliated adsorbents rely on physisorption, the higher capacities observed for KC/SP arise from its superior structural features—namely larger accessible surface area, better dispersion of active sites, and improved pore accessibility—not from stronger chemisorptive forces. This distinction is valuable because it confirms that KC/SP achieves high performance without relying on irreversible binding, further supporting its suitability for scalable and regenerable wastewater treatment applications.

#### 3.3.4 Advanced isotherm modeling

While classical adsorption isotherms—such as the Langmuir and Freundlich models—provide useful empirical descriptions of equilibrium behavior, they often fall short of explaining adsorption at a mechanistic or molecular level. Their primary limitation lies in the oversimplified treatment of adsorbent–adsorbate interactions, neglecting important factors such as steric constraints, energetic heterogeneity, receptor-site accessibility, and the multi-interaction nature of real-world adsorption systems (Ali et al., 2024; Gaber et al., 2025). These shortcomings have motivated the growing use of advanced, physics-based models that offer deeper insight into the thermodynamic and structural characteristics of adsorption.

In the present study, statistical-physics isotherm models were applied to elucidate the steric and energetic parameters governing phosphate adsorption onto KN/SP, U/SP, and KC/SP. Unlike conventional models, these advanced formulations enable the simultaneous assessment of how many phosphate ions can bind per receptor site (*n*), how densely these receptor sites are distributed within the adsorbent matrix (*N*_*m*_), and what the theoretical saturation capacity (*Q*_*sat*_) would be under idealized full-coverage conditions. These steric parameters provide a more accurate representation of the actual molecular packing and adsorption geometry on the modified serpentinite surfaces. In addition to steric considerations, several energetic parameters were evaluated to understand the thermodynamic driving forces of adsorption. These included the internal adsorption energy (*E*_*int*_), which reflects the strength of the adsorbate–adsorbent interaction at the local binding environment; adsorption entropy (*S*_*a*_), which provides insight into the degree of molecular disorder and structural rearrangement upon adsorption; the mean adsorption energy (*E*); and the enthalpy (G), which determines the spontaneity and feasibility of the overall process. Together, these parameters offer a multidimensional picture of phosphate binding behavior that cannot be captured by classical isotherm models.

**FIGURE 5:**
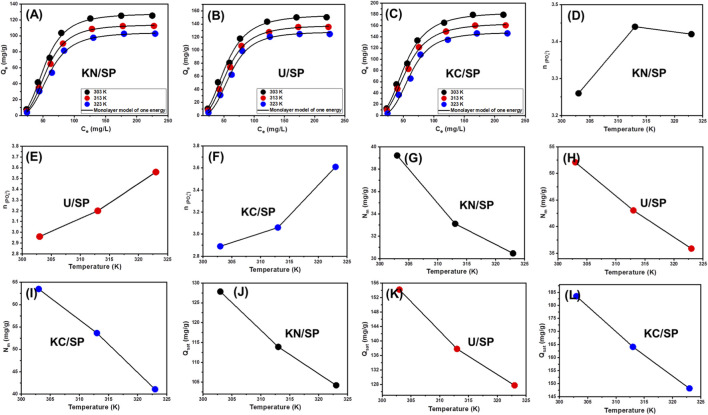
Shows fitting of the phosphate adsorption properties with advanced monolayer model with one energy site (KN/SP **(A)**, U/SP **(B)**, and KC/SP **(C)**) and the change in the steric parameters in terms of the operating temperature including number of adsorbed ions (KN/SP **(D)**, U/SP **(E)**, and KC/SP **(F)**), active sites density (KN/SP **(G)**, U/SP **(H)**, and KC/SP **(I)**), and saturation adsorption capacity (KN/SP **(J)**, U/SP **(K)**, and KC/SP **(L)**) (24 h contact time; 0.2 g/L dosage, 100 mL volume; and pH 6).

Nonlinear regression analysis was performed using the Levenberg–Marquardt optimization algorithm, ensuring robust parameter estimation and reliable convergence across the full concentration range. Based on the fitting quality and statistical indicators, the monolayer model with a uniform energy distribution across adsorption sites showed the highest consistency with the experimental data. This outcome suggests that the modified serpentinite materials possess energetically similar active sites and that phosphate adsorption proceeds predominantly *via* a single, well-defined interaction mechanism rather than a heterogeneous multilayer process. The fitted steric and energetic parameters, along with the goodness-of-fit metrics, are presented in Table 2 and visualized in Figures 5A–C.

Collectively, the success of the statistical-physics modeling provides strong evidence that the exfoliation treatments not only enhance the number of functional receptor sites but also create surfaces with uniform energetic characteristics. This uniformity explains the excellent agreement between the advanced monolayer model and experimental behavior, and it further underscores the mechanistic insight that phosphate adsorption onto KC/SP, U/SP, and KN/SP is dominated by highly accessible, energetically consistent binding sites formed through exfoliation-induced structural reorganization.

##### 3.3.4.1 Steric properties

###### 3.3.4.1.1 Number of adsorbed PO_4_^3-^ (*n*) per each site

The steric parameter n, representing the number of phosphate ions adsorbed per receptor site, provides critical insight into the adsorption orientation and molecular packing behavior of PO_4_^3-^ ions on KN/SP, U/SP, and KC/SP. This parameter reveals whether adsorption proceeds through simple one-to-one interactions or through more complex configurations such as multi-docking, multi-anchoring, or cooperative binding. In adsorption systems, *n* values below 1 typically correspond to horizontal or parallel alignment of phosphate ions on the adsorbent surface, implying monodentate or limited multidentate interactions. Conversely, *n* values greater than 1 reflect vertical, stacked, or non-parallel configurations, indicative of multi-ionic adsorption processes where several phosphate ions bind to a single receptor site through layered or clustered arrangements (Ali et al., 2024; Dhaouadi et al., 2021; Mobarak et al., 2021).

In this study, all three adsorbents exhibited n values exceeding 1, confirming that phosphate adsorption onto the exfoliated serpentinite-based materials is predominantly multi-ionic. For KN/SP, *n* ranged from 3.26 to 3.44 (Figure 5D; Table 2), suggesting that up to four phosphate ions can associate with a single active site. U/SP showed a comparable pattern with n values between 2.96 and 3.56 (Figure 5E; Table 2), while KC/SP displayed values from 3.06 to 3.61 (Figure 5F; Table 2). The consistently high *n* values across all materials highlight the significant structural modifications induced by exfoliation and intercalation treatments, which increase the accessibility, spacing, and reactivity of surface functional groups. These structural enhancements provide larger receptor pockets and more favorable geometric conditions that allow multiple PO_4_^3-^ ions to assemble around a single binding site.

The multi-ionic nature of adsorption indicated by *n* > 1 also implies that phosphate ions may interact cooperatively, forming vertically oriented molecular clusters stabilized by electrostatic attractions, hydrogen bonding, or surface-induced ordering. Such arrangements are characteristic of materials with high surface heterogeneity and abundant reactive domains created through chemical exfoliation. A clear upward trend in *n* values with increasing temperature (303–323K) was observed for all adsorbents. This temperature dependence suggests that higher thermal energy facilitates molecular mobility, reduces steric hindrance, and enhances the likelihood of phosphate aggregation or layered docking near active sites. The thermally activated increase in multi-docking behavior may stem from enhanced diffusion rates or partial weakening of hydration shells, which allow phosphate ions to pack more efficiently on the modified surfaces (Dhaouadi et al., 2021; Yang et al., 2022).

Overall, the steric parameter n provides strong mechanistic evidence that phosphate adsorption onto KN/SP, U/SP, and especially KC/SP proceeds through vertical, multi-layered, multi-molecular adsorption rather than simple monolayer uptake. These findings underscore the significant structural advantages introduced by exfoliation, which create expanded receptor environments capable of accommodating several phosphate ions per site—ultimately contributing to the high adsorption capacities observed for the modified serpentinite materials.

###### 3.3.4.1.2 Occupied active sites density (*N*_*m*_)

The occupied active site density (*N*_*m*_) represents the total number of accessible, energetically active adsorption sites available on the surfaces of KN/SP, U/SP, and KC/SP during phosphate uptake. This parameter reflects the structural quality of the adsorbent, including its degree of exfoliation, surface activation, and the extent to which functional groups are exposed and available for interaction with PO_4_^3-^ ions (Figures 5G–I; Table 2). For KN/SP, *N*_*m*_ values were 39.22 mg/g at 303 K, decreasing to 33.11 mg/g at 313 K and 30.47 mg/g at 323 K (Figure 5G). U/SP showed a similar but more pronounced trend, with *N*_*m*_ values of 52.09 mg/g at 303 K, 43.06 mg/g at 313 K, and 35.88 mg/g at 323 K (Figure 5H). KC/SP exhibited the highest site density among all materials, achieving 63.51 mg/g at 303 K, 53.63 mg/g at 313 K, and 41.05 mg/g at 323 K (Figure 5I).

The superior *N*_*m*_ values of KC/SP clearly demonstrate the effectiveness of CH_3_COOK-assisted exfoliation, which significantly enhances the number of available receptor sites, compared to KNO_3_-treated (KN/SP) and urea-modified (U/SP) materials. This enhancement is attributed to several structural improvements: a markedly increased surface area, greater exposure of Mg–OH and Si–OH functional groups, improved pore accessibility, and more efficient interlayer separation. Collectively, these modifications generate a larger population of high-quality binding sites capable of accommodating phosphate ions more effectively.

A consistent decline in *N*_*m*_ with rising temperature was observed for all adsorbents. This decrease suggests that higher temperatures reduce the number of accessible adsorption sites, likely through thermally induced structural relaxation, weakening of adsorbate–surface interactions, or partial desorption of pre-adsorbed species. The temperature sensitivity of *N*_*m*_ thus indicates that the surface activity and functional-group availability are governed by thermally responsive mechanisms (Dhaouadi et al., 2021; Diab et al., 2025b). Conversely, the steric coefficient *n* increased with temperature for all materials. This opposing trend between *N*_*m*_ and *n* reveals important mechanistic information: as the number of accessible sites decreases at higher temperatures, phosphate ions tend to pack more densely around the remaining available sites, resulting in enhanced multi-docking or vertical clustering behavior. This effect is consistent with thermally enhanced molecular mobility and increased PO_4_^3-^ aggregation, which promote multi-molecular interactions at fewer, but still reactive, sites.

The interplay between declining *N*_*m*_ and increasing *n* therefore reflects the dual influence of temperature on both the adsorbent and adsorbate. At higher temperature, phosphate transport is faster, but the accessible population of high-affinity sites may decrease if temperature alters surface hydroxylation/charge or interfacial water structure, thereby reducing net adsorption capacity (Budnyak et al., 2020). This dual behavior confirms that phosphate adsorption on the modified serpentinite materials exhibits thermally responsive steric and energetic characteristics, further highlighting the complex balance between surface structure, ionic aggregation behavior, and temperature-dependent adsorption dynamics.

###### 3.3.4.1.3 Adsorption capacity at the saturation state of (Q_sat_)

The saturation adsorption capacity (*Q*_*sat*_) represents the theoretical maximum amount of phosphate that KN/SP, U/SP, and KC/SP can retain under fully equilibrated conditions. This parameter integrates the contributions of both steric and surface-chemical properties by combining the density of active sites (*N*_*m*_) with the number of phosphate ions accommodated per site (*n*). Because *Q*_*sat*_ reflects the overall structural readiness of an adsorbent to bind and accumulate PO_4_^3-^ ions, it serves as a critical indicator of adsorption efficiency and material performance. Among the tested materials, KC/SP consistently demonstrated the highest saturation capacities, reaching 183.54 mg/g at 303 K, 164.10 mg/g at 313 K, and 148.19 mg/g at 323 K (Figure 5L; Table 2). These values reflect the extensive structural enhancements imparted by CH_3_COOK-assisted exfoliation, which noticeably increases the number of reactive surface sites, enlarges pore accessibility, and exposes a greater fraction of functional groups capable of anchoring phosphate. U/SP showed intermediate Q_sat_ values—154.18 mg/g, 137.79 mg/g, and 127.73 mg/g at the three respective temperatures (Figure 5K)—consistent with the moderate structural expansion achieved through urea intercalation. KN/SP produced the lowest _Qsat_ values of 127.85 mg/g, 113.89 mg/g, and 104.21 mg/g (Figure 5J), reflecting the comparatively weaker exfoliation and limited surface activation resulting from KNO_3_ treatment.

Across all adsorbents, *Q*_*sat*_ consistently decreased with increasing temperature, a pattern that underscores the exothermic nature of phosphate adsorption. As thermal energy rises, adsorbate–adsorbent interactions weaken, leading to reduced binding affinity and diminished loading at equilibrium. This thermal sensitivity is consistent with the physical adsorption–dominated mechanism identified earlier and reflects the reduced stability of weak electrostatic interactions at elevated temperatures. Notably, the temperature-driven decline in *Q*_*sat*_ aligns more strongly with reductions in *N*_*m*_ (site density) than with variations in *n* (number of ions per site). This relationship indicates that the overall adsorption capacity is more sensitive to the availability of accessible adsorption sites rather than the degree of multi-ionic occupation occurring at each site (Mobarak et al., 2021; Seifi et al., 2016). In other words, while multiple phosphate ions may continue to cluster around a single site at higher temperatures, the loss of total active sites due to thermally induced structural relaxation or reduced surface reactivity has a more pronounced impact on total adsorption potential (Budnyak et al., 2020; Dehbi et al., 2024; Kalaitzidou et al., 2022). Overall, the Q_sat_ trends reinforce the conclusion that adsorption efficiency is governed by the structural architecture of the exfoliated serpentinite materials. KC/SP, benefiting from the most extensive exfoliation and highest receptor-site density, consistently achieves the largest saturation capacities. These findings highlight the strong influence of modification chemistry on adsorbent design and underscore the importance of optimizing surface structure for high-capacity phosphate removal.

###### 3.3.4.1.4 Impact of the exfoliation mechanisms of the three reagents on steric parameters

**FIGURE 6:**
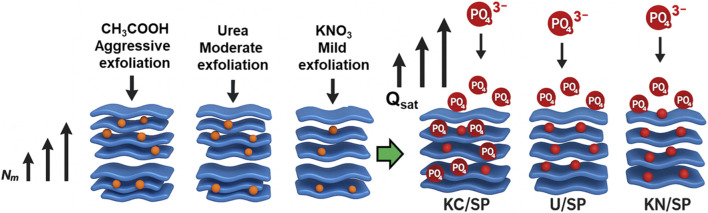
Schematic diagrams declare the impact of the exfoliation agents on the adsorption performances of the exfoliated products.

The exfoliation behavior of serpentine under the influence of CH_3_COOK, urea, and KNO_3_ plays a decisive role in determining the structural, steric, and energetic characteristics that ultimately control phosphate adsorption performance. Although all three reagents promote layer separation to some extent, their distinct intercalation chemistries give rise to markedly different structural transformations, affecting the density of adsorption sites (*N*_*m*_), the multi-molecular occupation per site (*n*), and the saturation capacity (*Q*_*sat*_) (Figure 6). These mechanistic differences account for the performance hierarchy observed among KC/SP, U/SP, and KN/SP. CH_3_COOK exhibits the most aggressive exfoliation capability due to the strong affinity of acetate ions for Mg-centered octahedral sheets in the serpentine structure. The acetate anion possesses both a carboxylate group capable of forming hydrogen bonds with surface hydroxyls and a relatively large ionic radius that introduces significant steric repulsion when inserted between layers (Ali et al., 2024; Diab et al., 2025a). This dual effect weakens interlayer hydrogen bonding and promotes deep intercalation, leading to substantial layer expansion, enhanced delamination, and the creation of abundant receptor sites with high accessibility. As a result, KC/SP consistently demonstrates the highest *N*_*m*_ values and the largest *Q*_*sat*_ values across all temperatures (Figure 6). The large number of active sites also encourages pronounced multi-anchoring and clustering effects, reflected in elevated n values. The stronger structural disruption induced by CH_3_COOK explains the superior adsorption capacity and the uniformity of energetically favorable binding sites across the KC/SP surface.

In contrast, urea induces exfoliation through hydrogen-bond donor–acceptor interactions. Upon heating, urea decomposes to species capable of forming multiple hydrogen bonds with the hydroxyl groups of serpentine, facilitating moderate disruption of the interlayer network (Ali et al., 2024; Seifi et al., 2016). This mechanism expands the interlayer spacing but to a lesser extent than acetate-assisted exfoliation, generating a balance between structural opening and preservation of some intrinsic crystallinity. Consequently, U/SP exhibits intermediate values of *N*_*m*_, reflecting a moderate density of exposed functional groups. The steric parameter n also remains above unity, indicating multi-molecular adsorption; however, its values are slightly lower than those of KC/SP due to more limited pore accessibility and weaker structural modification. Accordingly, U/SP demonstrates intermediate Q_sat_ values, consistent with its moderate exfoliation efficiency and adsorption-site density.

KNO_3_ operates primarily through ion-exchange interactions in which K^+^ replaces exchangeable cations near the sheet edges. Although nitrate is capable of weak interactions with interlayer hydroxyl groups, it lacks the strong hydrogen-bonding capability and steric bulk necessary to drive substantial delamination (Diab et al., 2025a). As a result, the exfoliation achieved in KN/SP is comparatively mild, and much of the layered structure remains intact. This limited structural disruption is directly reflected in the lower *N*_*m*_ values, indicating fewer accessible adsorption sites. Nevertheless, *n* values remain high, showing that even with limited exfoliation, phosphate ions can cluster around the available sites, forming vertical, multi-ion assemblies. However, due to the restricted number of these sites, KN/SP exhibits the lowest *Q*_*sat*_ values and the weakest adsorption capacity among the three modified samples.

Overall, the interplay between chemical functionality, ionic size, hydrogen-bonding ability, and interlayer interaction strength determines the exfoliation efficiency of the three reagents. CH_3_COOK produces the most extensive structural activation, resulting in high *N*_*m*_, elevated *n*, and superior *Q*_*sat*_ (Figure 6). Urea provides moderate exfoliation, yielding intermediate steric parameters and adsorption capacity. KNO_3_, with its limited structural impact, generates the lowest *N*_*m*_ and *Q*_*sat*_. These mechanistic differences highlight the crucial role of reagent-specific exfoliation pathways in tailoring the steric architecture and adsorption potential of serpentine-based adsorbents for efficient phosphate remediation.

##### 3.3.4.2 Energetic properties

###### 3.3.4.2.1 Adsorption energy and mechanism

Understanding the energetic characteristics of phosphate adsorption provides essential insight into the dominant interaction forces and enables differentiation between physical and chemical adsorption pathways. The adsorption energy change (ΔE) is a critical indicator in this context. In general, ΔE values below 40 kJ/mol are characteristic of physisorption, governed by weak intermolecular interactions such as hydrogen bonding (ΔE < 30 kJ/mol), dipole–dipole forces (2–29 kJ/mol), van der Waals attractions (4–10 kJ/mol), and electrostatic interactions (2–50 kJ/mol). In contrast, ΔE values exceeding 80 kJ/mol typically signify chemisorption involving covalent or valence-force interactions (Ali et al., 2021; Ashraf et al., 2022; Sellaoui et al., 2020). These thresholds serve as benchmarks for evaluating the nature and strength of adsorption mechanisms.

The ΔE values calculated for KN/SP, U/SP, and KC/SP, using Equation 5, ranged from −21.19 to −22.28 kJ/mol, −21.18 to −22.95 kJ/mol, and −21.17 to −22.89 kJ/mol, respectively (Table 2). These negative values confirm that phosphate adsorption onto all three materials is spontaneous, exothermic, and dominated by physisorption processes. However, while the magnitudes of ΔE classify the mechanism as physical, the origin and abundance of these interactions differ markedly depending on the exfoliation reagent and the functional groups exposed on the modified serpentine surface.

$$\Delta E=RT\;\ln\left(\frac{S}{C}\right)$$ (5)

**FIGURE 7:**
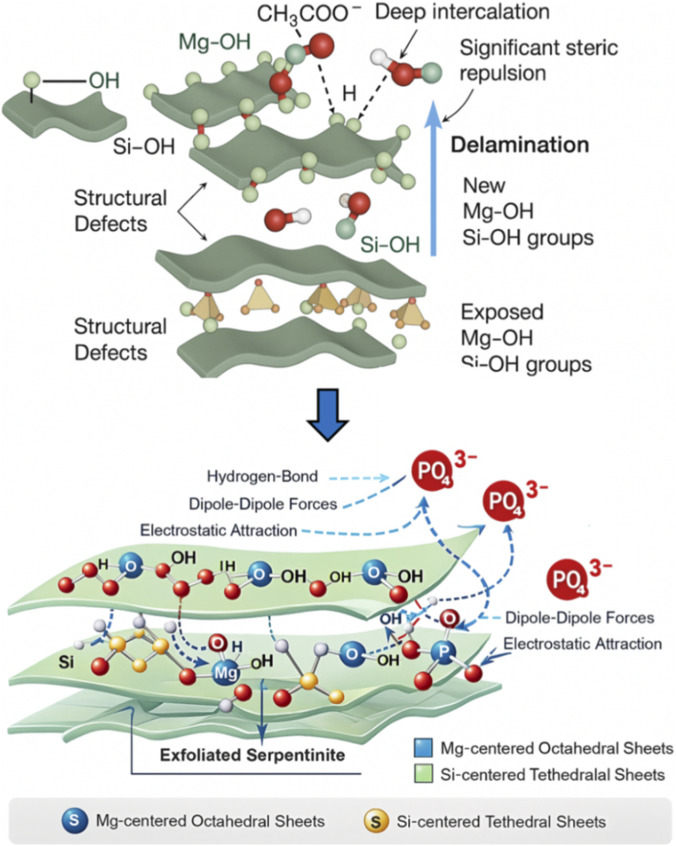
Schematic diagram for the adsorption mechanism of phosphate ions by exfoliated serpentinite lighting the impact of the exfoliation process on the exposure of the active sites.

Serpentinite naturally contains reactive surface groups—primarily Mg–OH, Si–OH, and structural defects generated at sheet edges. Exfoliation alters the number, distribution, and accessibility of these groups, thereby influencing the energetic landscape of adsorption (Figure 7). The acetate-assisted exfoliation (KC/SP) produces the most extensive structural activation. The CH_3_COO^−^ anion strongly interacts with Mg-centered octahedral sheets, forming hydrogen bonds with surface hydroxyls and generating significant steric repulsion within the interlayer region (Chen et al., 2024; Diab et al., 2025a; Makó et al., 2019; Shawky et al., 2019; Shemy et al., 2025; Wenk et al., 2025; Zheng and Lee, 2022; Zuo et al., 2017). This deep intercalation disrupts hydrogen-bond networks and induces pronounced delamination, exposing large numbers of Mg–OH and Si–OH groups and creating new defect sites (Figure 7).

Although the individual binding interactions remain within the physisorption energy range, KC/SP exhibits the highest density of reactive sites (*N*_*m*_) and supports extensive multi-molecular phosphate docking (high *n* values). This means that KC/SP does not rely on stronger interactions but rather on a larger number of energetically favorable adsorption events, mediated by through a combination of electrostatic attraction/outer-sphere association and hydrogen bonding, and—depending on solution chemistry—may also involve inner-sphere complexation (ligand exchange) with surface metal–OH groups (Gaber et al., 2025; Li et al., 2016; Li et al., 2023). This structural advantage explains why KC/SP consistently yields the highest *Q*_*sat*_ values despite having ΔE magnitudes comparable to U/SP and KN/SP.

The urea-modified sample (U/SP) exhibits moderate exfoliation driven by hydrogen-bond donor–acceptor interactions between urea and interlayer hydroxyl groups. Upon heating, urea decomposes into species capable of forming transient H-bond networks, partially widening interlayer spacing and enhancing surface polarity (Diab et al., 2025a; Seifi et al., 2016; Zhang et al., 2017). This exposes a substantial but more limited number of Mg–OH and Si–OH sites compared to KC/SP. As a result, U/SP displays intermediate *N*_*m*_ values and moderately high *n* values, supporting multi-ionic adsorption but with less accessibility and fewer defect-derived sites than KC/SP. The ΔE values for U/SP are similar in magnitude, consistent with adsorption dominated by H-bonding and electrostatic interactions, yet the structural framework limits its saturation capacity.

For KN/SP, nitrate intercalation induces only mild exfoliation. K^+^ ions participate in limited ion exchange at sheet edges, while NO_3_^−^—lacking strong hydrogen-bonding capability or significant steric bulk—produces minimal disruption of the interlayer hydrogen-bond network (Diab et al., 2025b). Consequently, KN/SP retains much of its original layered crystallinity, resulting in the lowest exposure of Mg–OH/Si–OH groups and the smallest *N*_*m*_ values. Nevertheless, the n values remain greater than unity, indicating that phosphate ions still form multi-ionic clusters around the few accessible sites. The weaker exfoliation, however, restricts the total number of adsorption events, leading to the lowest *Q*_*sat*_ among the three materials. The corresponding *ΔE* values reflect predominantly physical adsorption, governed by weaker electrostatic and dipole interactions due to the limited availability of reactive surface functionalities (Diab et al., 2025b). Overall, integrating the steric, structural, and energetic findings reveals the following hierarchy:

- KC/SP achieves the greatest adsorption performance due to extensive exfoliation, high *N*_*m*_, elevated *n* and dense networks of electrostatic attraction/outer-sphere association, hydrogen bonding, and dipolar interactions across newly generated surface sites.
- U/SP exhibits moderate structural activation with balanced exposure of functional groups, resulting in intermediate steric parameters and ΔE values characteristic of multi-site physisorption.
- KN/SP undergoes minimal interlayer disruption and displays the fewest accessible active sites, leading to the lowest *N*_*m*_ and *Q*_*sat*_, though the mechanism remains physisorptive.

Thus, while ΔE values confirm physical adsorption as the overarching mechanism, the exfoliation-driven structural evolution of the serpentine matrix governs the density, orientation, and accessibility of adsorption sites, which in turn determines the steric parameters and overall phosphate retention capacity. This integrated energetic–mechanistic understanding demonstrates that reagent-specific exfoliation pathways provide a powerful tool for tailoring the thermodynamic and structural behavior of serpentinite-based materials toward enhanced phosphate remediation.

###### 3.3.4.2.2 Thermodynamic functions

####### 3.3.4.2.2.1 Entropy

The entropy parameter (*S*_*a*_) associated with phosphate adsorption onto KN/SP, U/SP, and KC/SP provides a valuable window into the molecular organization, interfacial ordering, and dynamic behavior of these materials under different concentrations and temperatures. As a thermodynamic indicator, *S*_*a*_ reflects the extent to which adsorption induces structural rearrangement or restricts molecular mobility at the solid–solution interface (Dhaouadi et al., 2020; Gaber et al., 2025; Sellaoui et al., 2016). In this study, *S*_*a*_ was evaluated using the statistical-physics–based expression in Equation 6, which incorporates the density of adsorption sites (*N*_*m*_), the number of phosphate ions accommodated per site (n), and the half-saturation concentration (C_1_/_2_). Together, these parameters capture how steric architecture and the cooperative nature of multi-ion adsorption influence configurational entropy.

$$\frac{S_{a}}{K_{B}}=Nm\left\{\ln\left(1+{\left(\frac{C}{C_{\frac{1}{2}}}\right)}^{n}\right)-n{\left(\frac{C}{C_{\frac{1}{2}}}\right)}^{n}\;\frac{\ln\left(\frac{C}{C_{\frac{1}{2}}}\right)}{1+{\left(\frac{C}{C_{\frac{1}{2}}}\right)}^{n\;}}\;\right\}$$ (6)

**FIGURE 8:**
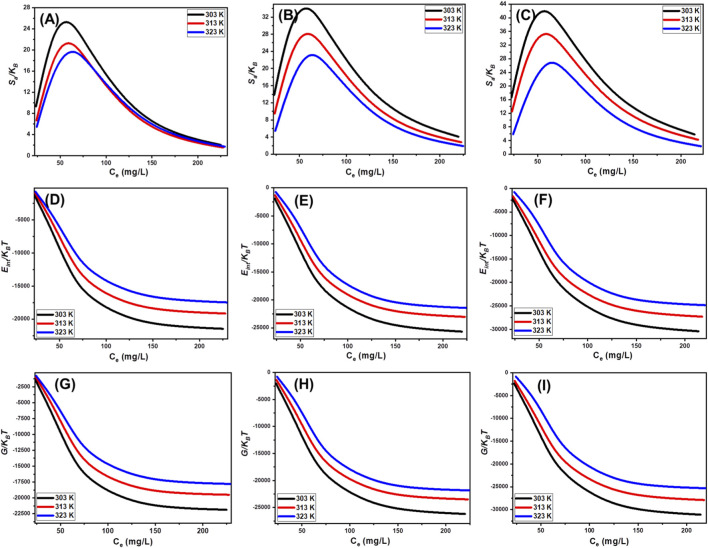
The change in the thermodynamic parameters in terms of the adsorption temperature including entropy (KN/SP **(A)**, U/SP **(B)**, and KC/SP **(C)**), internal energy (KN/SP **(D)**, U/SP **(E)**, and KC/SP **(F)**), and enthalpy (KN/SP **(G)**, U/SP **(H)**, and KC/SP **(I)**) (24 h contact time; 0.2 g/L dosage, 100 mL volume; and pH 6).

The entropy profiles derived from this model (Figures 8A–C) follow a distinct and interpretable pattern for all three adsorbents. At low phosphate concentrations, *S*_*a*_ increases noticeably, reflecting enhanced configurational freedom as phosphate ions initially approach and interact with available, high-accessibility Mg–OH and Si–OH surface groups. This early-stage rise in entropy is characteristic of systems where a large number of microstates remain accessible, and the docking of phosphate ions is not hindered by steric congestion. Such behavior is consistent with previous findings that the onset of adsorption into open, reactive nanostructures is accompanied by increased molecular disorder and interfacial fluctuation (Dhaouadi et al., 2020).

As phosphate concentration increases toward the half-saturation point, the entropy reaches a maximum. For KN/SP, peak Sa corresponds to equilibrium phosphate concentrations of 52.89 mg/L (303 K), 54.60 mg/L (313 K), and 59.20 mg/L (323 K) (Figure 8A), whereas U/SP exhibits maxima at 52.4 mg/L (303 K), 55.57 mg/L (313 K), and 59.3 mg/L (323 K) (Figure 8B). KC/SP displays slightly higher values, with peaks at 53.39 mg/L (303 K), 54.4 mg/L (313 K), and 61.6 mg/L (323 K) (Figure 8C). The convergence of these entropy maxima around C_1_/_2_ indicates that the configurational richness of the adsorption process is greatest when approximately half of the available receptor sites are occupied. At this stage, the system embodies a balance between free and occupied sites, and the probability distribution of adsorption configurations is widest.

Beyond the half-saturation region, however, *S*_*a*_ declines sharply with increasing phosphate concentration. This decrease indicates a progressive loss of randomness as receptor sites become filled and the adsorbate layer transitions from a dynamic partial-coverage regime to a more structurally ordered configuration. The decline in entropy signals cooperative constraints arising from steric crowding reduced diffusional freedom and the more fixed orientation of phosphate ions as they complete the surface monolayer or multi-layer docking permitted by the n > 1 steric behavior. In effect, the system undergoes an ordering transition, where additional adsorption reduces configurational diversity and drives the interface toward a more rigid, energetically stabilized arrangement (Sellaoui et al., 2016).

The observed entropy trends also correlate with the exfoliation efficiencies of the three materials. KC/SP, which possesses the greatest structural opening, highest receptor site density, and strongest steric multiplicity, exhibits the highest S_a_ maxima. This suggests that the extensive exposure of Mg–OH and Si–OH groups in KC/SP creates a more heterogeneous and flexible adsorption environment, allowing phosphate ions to explore a larger number of microstates before saturation. U/SP displays intermediate entropy behavior, consistent with its moderate degree of exfoliation, while KN/SP shows the lowest entropy values, reflecting its limited structural disruption and smaller population of available adsorption sites.

In summary, the entropy analysis reveals that phosphate adsorption onto KN/SP, U/SP, and KC/SP involves an initial regime dominated by configurational freedom and rapid docking, followed by a saturation-driven decrease in molecular disorder as adsorption sites are progressively filled. This evolution reflects both the steric crowding of the adsorbed layer and the substrate-induced reduction in molecular mobility within the modified serpentinite framework. The hierarchy in Sa values—KC/SP > U/SP > KN/SP—corresponds directly to differences in exfoliation intensity, site accessibility, and steric parameters, further demonstrating that structural activation of the serpentinite matrix governs not only the magnitude of adsorption but also the thermodynamic pathway through which adsorption proceeds.

####### 3.3.4.2.2.2 Internal energy and free enthalpy

The internal energy (*E*_*int*_) and free enthalpy (*G*) associated with phosphate adsorption onto KN/SP, U/SP, and KC/SP provide essential insight into the energetic pathways that govern the adsorption mechanism. Unlike classical thermodynamic treatments where *G* denotes Gibbs free energy, here *G* represents the statistical free enthalpy function derived from the advanced adsorption model. Both *E*_*int*_ and *G* serve as key indicators of how molecular interactions, site accessibility, and configurational ordering evolve across different phosphate concentrations and temperatures. These parameters were calculated using the statistical physics Equations 7, 8, which incorporate the adsorption site density (*N*_*m*_), the number of phosphate ions interacting with each site (n), the half-saturation concentration (C_1_/_2_), and the translational partition function (Zv) (Dehbi et al., 2024; Shi et al., 2022):

$$\frac{E_{\mathrm{int}}}{K_{B}T\;}=n\;N_{m}\left[\left(\;\frac{{\left(\frac{C}{C_{1/2}}\right)}^{n}\;\ln\left(\frac{C}{Z_{v}}\right)}{1+{\left(\frac{C}{C_{1/2}}\right)}^{n}}\right)-\left(\;\frac{n\ln\left(\frac{C}{C_{1/2}}\right)\;{\left(\frac{C}{C_{1/2}}\right)}^{n}}{1+{\left(\frac{C}{C_{1/2}}\right)}^{n}}\right)\right]$$ (7)

$$\frac{G}{K_{B}T\;}=n\;N_{m}\frac{\ln\left(\frac{C}{Z_{v}}\right)}{1+{\left(\frac{C_{1/2}}{C}\right)}^{n}}$$ (8)

Across all adsorbents, *E*_*int*_ exhibited consistently negative values that became increasingly negative with rising phosphate concentration, confirming that adsorption is governed by attractive interactions such as hydrogen bonding, dipole alignment, and electrostatic attraction between phosphate species (H_2_PO_4_^−^/HPO_4_^2-^) and the exposed Mg–OH and Si–OH groups on the exfoliated serpentinite surfaces. As temperature increased from 303 K to 323 K (Figures 8D–F), *E*_*int*_ values became less negative, indicating a gradual weakening of these attractive forces. This trend is characteristic of exothermic physisorption, where thermal agitation competes with the formation of stabilizing interfacial interactions (Shemy et al., 2025). The temperature-dependent change in *E*_*int*_ is also consistent with the enthalpy-related results shown in Figures 8G–I, confirming the thermodynamic coherence of the adsorption process.

The behavior of the free enthalpy G provides complementary information about the directionality and energetic drive of the adsorption process within the statistical-physics framework. For all three adsorbents, *G* remained negative across every temperature and concentration range examined (Figures 8G–I), indicating that adsorption is energetically favorable and naturally progressing under all conditions (Dhaouadi et al., 2021). The slight reduction in the magnitude of *G* at elevated temperatures reflects a reduction in the thermodynamic driving force of adsorption, yet the values remain sufficiently negative to ensure spontaneous uptake. This minor temperature effect aligns with the physical adsorption mechanism indicated by ΔE and entropy analyses, where adsorption arises from low-energy, reversible interactions rather than high-energy covalent bonding.

Together, *E*_*int*_ and G provide a unified energetic interpretation that dovetails with the steric and structural characteristics derived earlier. The strong negative *E*_*int*_ values at intermediate concentrations, followed by decreasing magnitude near saturation, reflect the increasing occupation of high-affinity Mg–OH and Si–OH sites and the concurrent reduction in configurational freedom as the surface becomes more ordered. Meanwhile, persistently negative free enthalpy values demonstrate that adsorption remains favorable even as steric crowding increases. These thermodynamic signatures confirm that phosphate retention on KN/SP, U/SP, and especially KC/SP is dominated by physisorption, governed by the availability of reactive surface groups, multi-ion docking (high n), and the density of accessible sites (*N*_*m*_), rather than by strong chemical bonding.

Overall, the internal energy and free enthalpy analyses reinforce that reagent-specific exfoliation pathways—particularly the deep intercalation and defect generation caused by CH_3_COO^−^ in KC/SP—control not only the steric parameters but also the energetic favorability of the adsorption process. This integrated thermodynamic–mechanistic understanding highlights why KC/SP exhibits the most efficient phosphate retention across the studied temperature range.

### 3.4 Effect of coexisting anions (SO_4_^2-^, NO_3_^−^, and HCO_3_^−^)

**FIGURE 9:**
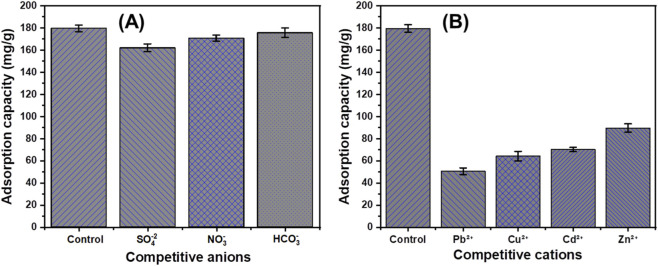
Effect of the coexisted anions **(A)** and metal cations **(B)** on the adsorption performances of KC/SP for phosphate ions (24 h contact time; 0.2 g/L dosage, 100 mL volume; and pH 6).

Wastewater streams rarely contain phosphate as a single contaminant; instead, they typically include a mixture of competing inorganic anions such as sulfate (SO_4_^2-^), nitrate (NO_3_^−^), and bicarbonate (HCO_3_^−^) (Figure 9A). Evaluating the adsorption behavior of KC/SP under such competitive conditions is therefore essential to assess its real applicability in complex environmental matrices. Owing to its extensive acetate-assisted exfoliation, KC/SP possesses the highest density of accessible adsorption sites (Nm = 63.51 mg/g) and the greatest saturation (Q_sat_ = 183.54 mg/g) and actual (179.4 mg/g) adsorption capacities, making it the most suitable material for examining the influence of coexisting ions on phosphate uptake.

The presence of sulfate had the strongest inhibitory effect on phosphate adsorption, decreasing the uptake from 179.4 mg/g to 161.8 mg/g (Figure 9A). This reduction reflects the divalent nature of SO_4_^2-^, which enables a stronger initial electrostatic attraction to positively charged surface regions. Nonetheless, sulfate interacts predominantly through outer-sphere mechanisms and exhibits low affinity to form the inner-sphere, multi-dentate complexes that phosphate establishes with Mg–OH and Si–OH groups (Hilbrandt et al., 2019; Wang et al., 2019). The multi-docking behavior of phosphate on KC/SP, supported by n > 3, allows phosphate ions to maintain preferential occupation of high-energy sites even when sulfate is present, thereby limiting the extent of competitive displacement. This observation signifies that the chemically activated surface of KC/SP is inherently tailored to favor phosphate coordination geometries over those of sulfate, ensuring sustained performance despite the presence of moderately competing anions.

Nitrate resulted in only a minor reduction of phosphate adsorption, reducing the capacity to 170.6 mg/g (Figure 9A). As a monovalent anion with low charge density and limited hydrogen-bonding potential, NO_3_^−^ exhibits weak interactions dominated by diffuse-layer electrostatics rather than site-specific binding (Heydari et al., 2024; Shi et al., 2022). Consequently, nitrate is unable to compete effectively with phosphate for the energetically uniform and highly accessible adsorption sites generated by exfoliation. The minimal interference observed in the presence of nitrate is particularly relevant for the treatment of agricultural drainage and mixed nutrient wastewaters, where high nitrate levels often coexist with phosphorus and may challenge conventional adsorbents. KC/SP, however, retains its phosphate selectivity and functional stability under such conditions.

Bicarbonate showed the weakest competitive effect among the tested anions, reducing phosphate adsorption only to 175.4 mg/g (Figure 9A). HCO_3_^−^ possesses high hydration energy and interacts weakly with solid surfaces, forming neither inner-sphere complexes nor directional bonds. Its influence is therefore limited to weak outer-layer electrostatic effects that do not significantly interfere with the strong hydrogen-bonded and electrostatically stabilized interactions driving phosphate adsorption onto KC/SP (Heydari et al., 2024; Shi et al., 2022). Maintaining high adsorption capacity in the presence of bicarbonate is particularly important in natural waters and municipal systems, where alkalinity is commonly elevated and often compromises the performance of many other adsorbents.

Overall, the influence of the coexisting anions on KC/SP follows the order SO_4_^2-^ > NO_3_^−^ > HCO_3_^−^, yet phosphate remains the overwhelmingly preferred species under all conditions. The consistently high capacities recorded in competitive systems demonstrate that the structural enhancements introduced by acetate exfoliation—especially the exposure of reactive Mg–OH and Si–OH groups and the generation of uniform, energetically favorable adsorption domains—play a decisive role in preserving phosphate selectivity. Importantly, the predominantly physisorptive nature of adsorption ensures rapid uptake, reversibility, and easy regeneration even in the presence of competing ions. These results collectively confirm that KC/SP is highly effective for phosphate removal in realistic wastewater environments containing multiple anionic species, reinforcing its potential for deployment in agricultural, municipal, and industrial treatment systems.

### 3.5 Effect of coexisting heavy metal cations (Pb^2+^, Cu^2+^, Cd^2+^, and Zn^2+^)

In addition to common inorganic anions, many industrial and agricultural wastewaters contain significant concentrations of heavy metal cations that may influence phosphate removal processes. The coexistence of Pb^2+^, Cu^2+^, Cd^2+^, and Zn^2+^ is particularly relevant in systems impacted by mining, electroplating, agricultural runoff, and fertilizer manufacturing. Therefore, evaluating the competitive effects of these cations on phosphate adsorption is essential for determining the operational robustness of the acetate-exfoliated serpentinite (KC/SP) under realistic wastewater conditions.

The presence of Pb^2+^ resulted in the strongest inhibitory effect on phosphate adsorption, reducing the uptake capacity from 179.4 mg/g to 50.7 mg/g (Figure 9B). This substantial decline reflects the exceptionally high affinity of Pb^2+^ for surface hydroxyl groups, as well as its strong tendency to form inner-sphere complexes with Mg–OH and Si–OH functionalities. Lead ions can effectively occupy high-energy adsorption domains that would otherwise serve as primary binding sites for phosphate, thereby restricting the number of available sites and suppressing competitive uptake. The formation of stable Pb–OH surface complexes limits the surface accessibility of phosphate, explaining the pronounced reduction in capacity (Cao et al., 2019; Miao et al., 2024).

Cu^2+^ also exhibited a considerable competitive effect, lowering phosphate adsorption to 64.3 mg/g (Figure 9B). Copper ions possess strong polarizing ability and a high propensity for forming bidentate and tridentate coordination structures with oxygen-containing functional groups. As a result, Cu^2+^ is capable of partially replacing phosphate at key reactive sites, despite the strong multi-molecular adsorption mechanism favored by phosphate on KC/SP (Kocik et al., 2024; Szewczuk-Karpisz et al., 2023). Although this competitive effect is less severe than that of Pb^2+^, it nonetheless highlights the need to account for copper contamination when evaluating KC/SP performance in metalliferous wastewaters.

The presence of Cd^2+^ caused a moderate suppression of phosphate uptake, with the capacity decreasing to 70.2 mg/g. Cadmium forms relatively weaker surface complexes compared to Pb^2+^ and Cu^2+^, owing to its lower electronegativity and less favorable hydrolysis behavior (Pham et al., 2021; Xue et al., 2023; Zhao et al., 2024). However, its divalent charge still enables partial competition for positively charged or neutral hydroxyl groups, particularly at defect-rich regions generated by exfoliation. The reduction observed reflects moderate competitive binding without complete displacement of phosphate from high-affinity sites.

Zn^2+^ produced the weakest competitive effect among the tested cations, reducing the phosphate adsorption capacity to 89.7 mg/g. Zinc has comparatively weaker surface binding tendencies and a strong preference for remaining in hydrated aqueous complexes. As such, Zn^2+^ only partially interferes with phosphate adsorption on KC/SP (Duan and Fedler, 2022; Li et al., 2024). The minimal inhibition observed confirms that KC/SP can effectively remove phosphate in zinc-rich waters, such as agricultural runoff and fertilizer effluents.

Overall, the inhibition trend for the examined cations follows the order: Pb^2+^ > Cu^2+^ > Cd^2+^ > Zn^2+^, corresponding closely to their relative binding strengths and coordination tendencies with surface hydroxyl groups. Despite these competitive effects, KC/SP maintains substantial adsorption capacity in all cases, demonstrating its resilience and suitability for phosphate remediation in complex wastewater environments. The results highlight the importance of considering metal–surface interactions in systems where heavy metals coexist with phosphate, and they further emphasize the structural advantages of KC/SP in maintaining high performance under challenging multicomponent conditions.

### 3.6 Recyclability

The long-term economic feasibility and practical deployment of KC/SP as a phosphate adsorbent depend largely on its durability and ability to withstand multiple adsorption–desorption cycles. Recyclability is a critical requirement for any sorbent intended for real wastewater treatment, as it directly influences operational costs, sustainability, and material efficiency. To assess the stability of KC/SP under repeated use, a multi-cycle regeneration test was performed under stringent experimental conditions representing the highest phosphate concentrations evaluated in this study. After each adsorption cycle, the spent KC/SP was regenerated through a standardized washing protocol consisting of gentle mechanical agitation for 30 min, followed by rinsing with distilled water to remove residual alkaline species. The cleaned material was subsequently dried at 50 °C for 12 h to restore its structural integrity before reuse. This method aligns with commonly practiced regeneration approaches for mineral-based adsorbents, ensuring minimal structural alteration while effectively desorbing previously bound phosphate.

**FIGURE 10:**
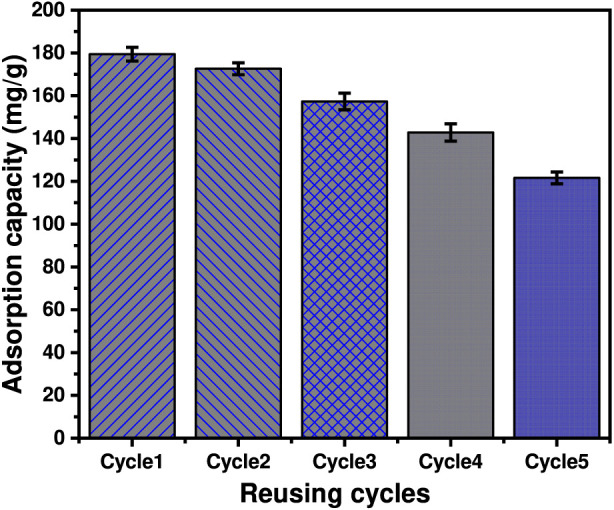
The recyclability properties of KC/SP as adsorbent for phosphate ions (24 h contact time; 0.2 g/L dosage, 100 mL volume; and pH 6).

The regenerated KC/SP was subjected to five consecutive adsorption cycles, each conducted with 100 mL of phosphate solution at 250 mg/L, pH 6, a sorbent dosage of 0.2 g/L, contact time of 24 h, and a temperature of 303 K. These conditions reflect an intentionally challenging scenario designed to evaluate material resilience under high pollutant loading. The results revealed that KC/SP preserved a substantial proportion of its adsorption capacity across the five cycles. Specifically, phosphate uptake reached 179.4 mg/g, 172.6 mg/g, 157.3 mg/g, 142.8 mg/g, and 121.6 mg/g in cycles 1 through 5, respectively (Figure 10). Although a gradual decline in performance was observed, the high retention performances even after five cycles reflects a strong structural robustness and a stable adsorption mechanism. The observed decrease can be attributed to the progressive accumulation of strongly bound phosphate species and the partial saturation or blockage of high-affinity active sites. As surface functional groups participate in repeated interactions with phosphate ions, some binding environments evolve into stable inner-sphere complexes that are less easily desorbed during regeneration. This phenomenon is typical for layered silicates and magnesium-rich phyllosilicates, where reactive hydroxyl-rich sites gradually undergo surface coordination modifications with repeated cycles.

Despite this decline, the capacity remaining after the fifth cycle remains significantly higher than many commercially used adsorbents and far exceeds the required capacity for practical phosphate removal at environmentally relevant concentrations. This demonstrates that KC/SP maintains its functional performance and structural stability even under aggressive regeneration and high-concentration stress conditions. The strong retention behavior highlights its suitability for repeated deployment in continuous or semi-continuous treatment systems, further reinforcing its applicability for sustainable phosphate remediation in real-world water bodies such as Lake Qarun.

### 3.7 Realistic remediation of phosphate-contaminated water from Lake Qarun (Fayum, Egypt)

Lake Qarun, located in the Fayum Depression of Egypt, is one of the most environmentally stressed inland lakes in North Africa. Over the past decades, the lake has undergone severe ecological degradation due to the continuous discharge of agricultural drainage, wastewater effluents, and fish-farm residues. These uncontrolled inputs have dramatically altered the lake’s hydrochemistry, increasing its salinity and converting it into a chloride–sulfate dominated water body with exceptionally high ionic strength. The major cations (Na^+^, K^+^, Mg^2+^, Ca^2+^) and anions (Cl^−^, SO_4_^2-^, HCO_3_^−^) occur at concentrations far exceeding those typical of freshwater systems. In particular, the substantial inflow of nutrient-rich agricultural drainage has introduced elevated levels of dissolved phosphate, which is a key driver of the lake’s chronic eutrophication.

Phosphate enrichment has contributed significantly to excessive algal blooms, depletion of dissolved oxygen, and recurrent fish mortality events within Lake Qarun. Long-term eutrophication has also resulted in the accumulation of organic matter, increased turbidity, habitat degradation, and sharp declines in biodiversity among fish, mollusks, and benthic invertebrates. Elevated phosphate concentrations, even at levels as low as 0.3–0.5 mg/L, can trigger eutrophic conditions in closed or semi-closed water bodies. With measured phosphate levels in Lake Qarun often reaching 0.6–1.0 mg/L near agricultural discharge points, mitigation of phosphate is essential for ecological restoration and to suppress harmful algal proliferation.

In this study, a realistic remediation experiment was designed to evaluate the performance of the acetate-exfoliated serpentinite (KC/SP) in treating phosphate-contaminated Lake Qarun water. A representative water sample containing 0.63 mg/L phosphate was treated using 1.0 g of KC/SP per 1000 mL under a 180-min contact period, reflecting practical operating conditions relevant for real-world remediation units. The treatment was conducted without altering the natural ionic composition of the lake water, thereby preserving its high salinity, elevated sulfate concentration, and the presence of trace metal ions such as Fe, Mn, Zn, Cu, and Pb, which could potentially influence adsorption processes.

Following treatment, the phosphate concentration in the lake-water sample decreased dramatically to below 0.002 mg/L, indicating near-complete removal and achieving levels well below the eutrophication threshold (<0.05 mg/L) recommended for freshwater ecological stability. The significant reduction confirms that KC/SP retains exceptional adsorption capability even when exposed to the highly competitive ionic matrix of Lake Qarun. This performance reflects the high active-site density and multi-ionic phosphate docking behavior (n > 3) characteristic of KC/SP, enabling it to overcome ionic interference from Na^+^, Mg^2+^, Ca^2+^, sulfate, bicarbonate, and naturally occurring trace metals.

The obtained removal efficiency indicates that the current conditions (1 g per 1 L, 180 min) provide substantial overcapacity for phosphate adsorption. However, the system can be further enhanced by increasing the contact time, increasing the dosage, or implementing sequential treatment cycles. Integrating two or three remediation cycles would completely eliminate any residual phosphorus and maintain concentrations well below ecological risk levels, ensuring long-term stability and reducing the likelihood of algal regrowth. Assessment of the treated water demonstrates that phosphate levels after remediation are consistent with international standards for preventing eutrophication and are appropriate for aquatic ecosystem recovery. The substantial reduction in phosphorus load is expected to lower algal biomass, improve water clarity, and support the reoxygenation and rehabilitation of aquatic habitats. Because KC/SP relies on a predominantly physisorption-driven mechanism, regeneration of the spent material is feasible, making it suitable for scalable or decentralized treatment systems in the Fayum region.

In summary, under realistic field conditions, KC/SP provides highly efficient removal of phosphate from Lake Qarun water despite extreme salinity, elevated sulfate levels, and trace-metal presence. The reduction of phosphate to <0.002 mg/L demonstrates the strong potential of KC/SP as a practical and effective remediation technology capable of significantly mitigating eutrophication and contributing to the ecological restoration of Lake Qarun.

### 3.8 Comparison study

A comparative evaluation was conducted to position KC/SP within the broader landscape of phosphate-removal technologies and to benchmark its performance against raw serpentinite and a wide range of conventional and advanced adsorbents. The findings clearly demonstrate that KC/SP exhibits a superior adsorption capability, achieving a maximum phosphate uptake (Q_max_) of 183.5 mg/g, which is approximately three times higher than that of unmodified serpentinite (61.2 mg/g). This substantial improvement underscores the effectiveness of the acetate-assisted exfoliation process in enhancing the structural accessibility and chemical reactivity of serpentinite.

When compared with traditional mineral adsorbents (e.g., zeolites, clays, and LDHs), carbonaceous sorbents (biochars, GO-based hybrids), and metal–organic frameworks (MOFs), KC/SP consistently outperforms these materials by a considerable margin. Many commercial or synthetic adsorbents—such as zeolite A (52.91 mg/g), calcined Mg–Al LDHs (40.78 mg/g), La-modified SBA-15 (45.6 mg/g), and titanium-modified zeolites (37.60 mg/g)—exhibit significantly lower capacities, often requiring higher dosages or extended contact times to achieve meaningful removal. Even advanced nanostructured materials, including zirconia nanoparticles (99 mg/g), lanthanum hydroxides (107.5 mg/g), and La-doped graphene composites (116.28 mg/g), fall short of the performance achieved by KC/SP.

The exceptional adsorption efficiency of KC/SP can be attributed to several synergistic factors arising from the exfoliation treatment. First, the delamination process greatly increases the accessibility of Mg–OH and Si–OH surface groups, which serve as the primary phosphate-binding domains. Second, the structural expansion and creation of high-energy adsorption sites enable multi-molecular uptake, as reflected by the high steric occupancy parameter (n > 3) obtained from the statistical-physics analysis. Third, the uniform distribution of reactive sites across the exfoliated layers promotes both rapid diffusion and enhanced surface interactions, leading to markedly improved kinetics compared with other adsorbents.

Another notable advantage of KC/SP is its low required dosage. Because of its high surface reactivity and energetically favorable adsorption domains, smaller quantities of KC/SP can achieve the same or higher removal efficiencies compared with substantially larger dosages of conventional materials. This translates into major economic benefits, including reduced material consumption, lower regeneration costs, and decreased environmental impact during production and disposal. Furthermore, unlike synthetic adsorbents that often depend on multi-step chemical treatments, KC/SP is derived from a naturally abundant serpentinite mineral, offering a sustainable and cost-effective option for large-scale applications.

Collectively, these comparisons reveal that KC/SP stands among the highest-performing phosphate adsorbents reported to date, combining high efficiency, scalability, rapid kinetics, and economic practicality. Its outstanding performance across a wide range of competing materials reinforces its suitability for advanced wastewater treatment systems, agricultural drainage remediation, and eutrophication control in natural water bodies.

## 4 Conclusion

This study demonstrates that intercalation-driven exfoliation of serpentinite represents a highly effective and realistic strategy for phosphate removal from aqueous systems. Among the investigated materials, potassium acetate–modified serpentinite (KC/SP) exhibited the highest adsorption performance, achieving a maximum phosphate uptake of 183.54 mg/g, significantly outperforming U/SP (154.18 mg/g) and KN/SP (127.85 mg/g). The enhanced performance of KC/SP is attributed to its superior exfoliation degree, higher density of accessible active sites (up to 63.51 mg/g), and favorable surface chemistry that promotes strong yet reversible interactions with phosphate species.

Kinetic and equilibrium analyses confirmed that phosphate adsorption proceeds *via* a rapid, surface-controlled process, best described by the pseudo-first-order kinetic model and Langmuir isotherm, respectively. Statistical physics modeling further revealed a multisite adsorption mechanism (n ≈ 4) governed primarily by physical interactions—electrostatic attraction, hydrogen bonding, and ion–dipole forces—resulting in adsorption energies below 25 kJ/mol. These features explain both the high uptake capacity and the favorable regenerability of the adsorbent. Importantly, adsorption performance remained stable in the presence of competing anions and metal ions, and was successfully validated using real Lake Qarun water, confirming the material’s robustness under chemically complex and saline conditions.

From a practical perspective, the results highlight the strong potential of KC/SP as a low-cost, scalable, and environmentally benign adsorbent for phosphate remediation in agricultural runoff, industrial effluents, and eutrophic water bodies. Nevertheless, further work is recommended to assess long-term cyclic stability, regeneration efficiency over multiple adsorptions–desorption cycles, and performance under continuous-flow conditions. Future studies should also evaluate techno-economic feasibility and life-cycle impacts to support the transition of this material from laboratory-scale development to real-world implementation.

## Data Availability

The original contributions presented in the study are included in the article/Supplementary Material, further inquiries can be directed to the corresponding author.
