# Supplementary material for: Realistic and sustainable phosphate adsorption using intercalant-engineered exfoliated serpentinite: mechanistic insights and real-water validation
Source: Front Chem. 2026 Mar 27;14:1791565. doi: 10.3389/fchem.2026.1791565 (PMC13066218; doi:10.3389/fchem.2026.1791565)
Supplement: Supplementary file 1 [file Table1.docx]

Table.S1. Nonlinear equations of kinetic, classic isotherm, and advanced isotherm models

| Kinetic models | | |
| --- | --- | --- |
| Model | Equation | Parameters |
| Pseudo-first-order | $Q_{t}=Q_{e} (1-e^{{-k}_{1}.t})$ | Q_t_ (mg/g) is the adsorbed ions at time (t), and K_1_ is the rate constant of the first-order adsorption (1/min) |
| Pseudo-second-order | $Q_{t}=\frac{Q_{e}^{2}k_{2}t}{1+Q_{e}k_{2}t}$ | Qe is the quantity of adsorbed ions after equilibration (mg/g), and K_2_ is the model rate constant (g/mg min). |
| Classic Isotherm models | | |
| Model | Equation | Parameters |
| Langmuir | $Q_{e}=\frac{Q_{max} bC_{e}}{(1+bC_{e})}$ | *C_e_* is the rest ions concentrations (mg/L), *Q_max_* is the theoritical maximum adsorption capacity (mg/g), and *b* is the Langmuir constant (L/mg) |
| Freundlich | $Q_{e}=K_{f}C_{e}^{1/n}$ | K_F_ (mg/g) is the constant of Freundlich model related to the adsorption capacity and n is the constant of Freundlich model related to the adsorption intensities |
| Dubinin–Radushkevich | $Q_{e}=Q_{m}e^{-\betaɛ^{2}}$ | β (mol^2^/KJ^2^) is the D-R constant, ɛ (KJ^2^/mol^2^) is the Polanyi potential, and Q_m_ is the adsorption capacity (mg/g) |
| Advanced isotherm models | | |
| Model | Equation | Parameters |
| Monolayer model with one energy site (Model 1) | $Q=nN_{o} =\frac{nN_{M}}{1+{(\frac{C1/2}{C})}^{n}}=\frac{Q_{o}}{1+{(\frac{C1/2}{C})}^{n}}$ | Q is the adsorbed quantities in mg/g  n is the number of adsorbed ion per site  Nm is the density of the effective receptor sites (mg/g)  Q_o_ is the adsorption capacity at the saturation state in mg/g  C1/2 is the concentration of the ions at half saturation stage in mg/L  C1 and C2 are the concentrations of the ions at the half saturation stage for the first active sites and the second active sites, respectively  n1 and n2 are the adsorbed ions per site for the first active sites and the second active sites, respectively |
| Monolayer model with two energy sites (Model 2) | $Q=\frac{n_{1}N_{1M}}{1+{(\frac{C_{1}}{C})}^{n_{1}}}+\frac{n_{2}N_{2M}}{1+{(\frac{C_{2}}{C})}^{n_{2}}}$ |  |
| Double layer model with one energy site (Model 3) | $Q=Q_{o}\frac{({\frac{C}{C1/2})}^{n}+2({\frac{C}{C1/2})}^{2n}}{1+({\frac{C}{C1/2})}^{n}+({\frac{C}{C1/2})}^{2n}}$ |  |
| Double layer model with two energy sites (Model 3) | $Q=Q_{o}\frac{({\frac{C}{C1})}^{n}+2({\frac{C}{C2})}^{2n}}{1+({\frac{C}{C1})}^{n}+({\frac{C}{C2})}^{2n}}$ |  |

**Table.S2.** Comparison study between the developed KC/SP as adsorbent and other adsorbents in literature

| Adsorbent | Q_max (mg/g) | Reference |
| --- | --- | --- |
| Hydrous zirconium oxide | 51.8 | Lin et al. (2017) |
| Zeolite A | 52.91 | Hamdi & Srasra (2012) |
| Titania/GO | 33.11 | Sakulpaisan et al. (2016) |
| Mg/Al LDH–modified biochar | 56.12 | Deng et al. (2021) |
| La-doping magnetic graphene | 116.28 | Nodeh et al. (2017) |
| Calcined Mg–Al LDHs | 40.78 | Das et al. (2006) |
| MCM-41/Rice husk | 21 | Seliem et al. (2016) |
| Titanium-modified zeolite | 37.60 | Alshameri et al. (2014) |
| Zirconia/graphite oxide | 149.3 | Zong et al. (2013) |
| LTA MOFs | 62.8 | Kumar et al. (2021) |
| Biochar | 133 | Yao et al. (2011) |
| Mg(OH)₂/ZrO₂ | 87.2 | Lin et al. (2019) |
| Kaolinitic clay | 38.46 | Hamdi & Srasra (2012) |
| Lanthanum hydroxides | 107.5 | Xie et al. (2014) |
| ZrO₂ nanoparticles | 99 | Su et al. (2013) |
| Al-modified biochar | 57.49 | Yin et al. (2018) |
| La100SBA-15 | 45.6 | Yang et al. (2011) |
| KC/SP | **183.5** | **This study** |
